# Automated extraction of the arterial input function from brain images for parametric PET studies

**DOI:** 10.1186/s13550-024-01100-x

**Published:** 2024-04-01

**Authors:** Hamed Moradi, Rajat Vashistha, Soumen Ghosh, Kieran O’Brien, Amanda Hammond, Axel Rominger, Hasan Sari, Kuangyu Shi, Viktor Vegh, David Reutens

**Affiliations:** 1 Centre for Advanced Imaging, Australian Institute for Bioengineering and Nanotechnology, The University of Queensland, Brisbane, Australia; 2 ARC Training Centre for Innovation in Biomedical Imaging Technology, The University of Queensland, Brisbane, Australia; 3 Siemens Healthcare Pty Ltd, Melbourne, Australia; 4 Department of Nuclear Medicine, Inselspital, Bern University Hospital, University of Bern, Freiburgstrasse 18, 3010 Bern, Switzerland; 5 Advanced Clinical Imaging Technology, Siemens Healthcare AG, Lausanne, Switzerland

**Keywords:** Dynamic PET, Parametric mapping, Non-invasive arterial input function, Automatic AIF estimation

## Abstract

**Background:**

Accurate measurement of the arterial input function (AIF) is crucial for parametric PET studies, but the AIF is commonly derived from invasive arterial blood sampling. It is possible to use an image-derived input function (IDIF) obtained by imaging a large blood pool, but IDIF measurement in PET brain studies performed on standard field of view scanners is challenging due to lack of a large blood pool in the field-of-view. Here we describe a novel automated approach to estimate the AIF from brain images.

**Results:**

Total body ^18^F-FDG PET data from 12 subjects were split into a model adjustment group (n = 6) and a validation group (n = 6). We developed an AIF estimation framework using wavelet-based methods and unsupervised machine learning to distinguish arterial and venous activity curves, compared to the IDIF from the descending aorta. All of the automatically extracted AIFs in the validation group had similar shape to the IDIF derived from the descending aorta IDIF. The average area under the curve error and normalised root mean square error across validation data were − 1.59 ± 2.93% and 0.17 ± 0.07.

**Conclusions:**

Our automated AIF framework accurately estimates the AIF from brain images. It reduces operator-dependence, and could facilitate the clinical adoption of parametric PET.

**Supplementary Information:**

The online version contains supplementary material available at 10.1186/s13550-024-01100-x.

## Background

Positron emission tomography (PET) using ^18^F-fluorodeoxyglucose (^18^F-FDG) has established as a robust diagnostic tool, Offering unique insights into tissue and organ metabolism [1]

The integration of dynamic PET studies with kinetic modeling techniques provides valuable insights into the physiological aspects of PET tracer dynamics. This approach yields biologically-based parameters at the level of individual voxels or regions of interest (ROIs), capturing crucial information on tracer delivery, metabolism, and binding characteristics [2]. In the case of ^18^F-FDG, parametric PET generates detailed images of kinetic parameters at the voxel level, explaining ^18^F-FDG uptake based on temporal changes in tissue tracer concentration extracted from dynamic PET data [3]. A standard method for kinetic parameter estimation involves utilizing a compartment model, originally developed by Sokoloff et al. [4]. This model allows for the estimation of key kinetic parameters, including ${K}_{1}$ and ${k}_{2}$ (the influx and efflux rates of the tracer between blood and tissue), and ${k}_{3}$ and ${k}_{4}$ (the phosphorylation and dephosphorylation rates of ^18^F-FDG). The net influx rate, ${K}_{i}={K}_{1}{k}_{3}/{k}_{2}+{k}_{3}$, provides an overall measure of tissue tracer uptake [4–6].

Interest in parametric PET is growing due to the increase of interest in precision medicine and parametric images are used in diagnosis, treatment monitoring, and to determine prognosis, particularly in neurological diseases and oncology. This technique holds the promise of delivering more comprehensive clinical diagnostic information compared to current SUV-based methods [7].

Parametric PET requires the accurate estimation of the arterial input function (AIF), which characterizes the time-dependent changes of tracer concentration in the arterial blood pool. Conventionally, the AIF is measured using arterial blood samples, a method which is time-consuming and invasive, with the potential for significant complications [7].

To enable parametric PET imaging without arterial sampling, several non-invasive alternatives have been proposed: population-based input functions; joint estimation of AIF with the kinetic parameters; and image-derived input functions (IDIF). The population-based input function methods are simple to apply, but unfortunately introduce errors due to inter-subject physiological variability and variations in injection protocols [8]. Conversely the joint estimation of the AIF with the kinetic parameters increases the unknowns required in the kinetic model and therefore is prone to overfitting [9, 10].

The use of an IDIF, which involves estimating the AIF directly from PET images, is an attractive non-invasive alternative to arterial sampling. The IDIF relies on the presence of suitable artery within the field of view and has been validated for blood pools such as the heart [11], aorta [12], and femoral arteries [13]. The large size of which facilitates the placement of a region of interest (ROI) and correction or even omission of corrections for the partial volume effect [2, 3, 14–17].

In PET brain studies using clinical standard field-of-view scanners and single-bed protocols, accurate IDIF estimation is still challenging as the images lack large blood pools. The AIF extracted from intracranial vessels in PET images is impacted by partial volume effect caused by the small size of the vessels compared to the limited spatial resolution of PET scanners [18, 19]. These issues may lead to underestimation of the AIF, affecting its waveform [15, 16]. A study using the HRRT PET system (~ 3 mm resolution) proposed a multimodal approach to generate IDIF curves, comparing them with blood sampling and evaluating MR registration. Without MR registration, notable underestimation occurred, with an AUC ratio of 0.40 ± 0.19. Combining PET with MR segmented regions improves results compared to PET alone, yet some underestimation persists, as evidenced by an increased AUC ratio to 0.69 ± 0.26 [20].

Furthermore, current IDIF approaches require ROIs to be manual positioned over the internal carotid arteries or venous sinuses, which is both time-consuming and operator dependent [15].

To enhance the accuracy of estimating IDIF from brain images, one approach involves outlining the carotid arteries using high-resolution MRI and co-registering the MRI to PET images [15, 21, 22]. While this method demonstrates good agreement with gold standard techniques [18], it requires an additional MRI and involves complex segmentation and co-registration pipelines [23, 24], or may be not practical in certain cohorts [25–27].

Atlas-based methods for IDIF estimation, which do not require individual additional MR images and instead rely on predefined blood vessels identified from the MR template [22, 28, 29], may encounter challenges such as co-registration errors and an inability to account for subject-specific variations [15].

Alternatively, automated and semi-automated AIF extraction methods have been proposed. For example, clustered-component analysis, grouping voxels with similar time-activity curves for AIF extraction [30, 31], holds potential for automated AIF estimation. These automated and semi-automated techniques require preselection of image classes and advanced partial volume correction [16, 30–33].

More recently, machine learning has been employed for tissue segmentation and AIF extraction. Kuttner et al. [34] demonstrated that long short-term memory (LSTM) recurrent neural network models produce lower error rates than Gaussian process regression for the estimation of the input function from tissue time activity curves. Varny et al. utilized a deep neural network implementation to estimate AIF using sinogram data [35]. However, a drawback of current machine learning methods is their requirement for computational resources and for extensive training data.

We aimed to develop an automated non-invasive method for accurately estimating the AIF using PET brain images alone, without modifying the standard data acquisition process. By combining similarity metrics with unsupervised machine learning, we differentiated between arteries and veins, enabling precise AIF estimation comparable to IDIFs from large blood pools. Validation was performed using dynamic PET data from a long axial field of view scanner, allowing comparison of the brain-extracted AIF with the IDIF obtained from the large blood pool in the same field of view.

## Materials and methods

### Human PET imaging—study participants

This study involved 12 subjects who were oncological patients (4 females and 8 males) with various tumor types, a mean age of 62 ± 16 years and a mean weight of 82 ± 19 kg. Data obtained from a prior study, which received approval from the local institutional review board at the Department of Nuclear Medicine, Inselspital, Bern University Hospital, University of Bern (KEK 2019–02,193), were made available for this study [2]. Subjects were randomly assigned to a model adjustment group (n = 6) and a validation group (n = 6) see Additional file 1: Table S1 for details).

### PET-CT data acquisition

Total Body PET data were acquired using a Biograph Vision Quadra PET/CT (Siemens Healthineers) system with a 106 cm axial field-of-view. In-plane spatial resolution was 3.27 mm full-width at half-maximum (FWHM) [36]. List-mode acquisition commenced 15 s before the intravenous bolus injection of ^18^F-FDG (average activity: 250 ± 58 MBq), followed by a 50mL saline flush. Data were collected for 65 min and partitioned into 62 frames with durations of 2 × 10 s, 30 × 2 s, 4 × 10 s, 8 × 30 s, 4 × 60 s, 5 × 120 s, and 9 × 300 s. The images were reconstructed and then smoothed using a 2 mm FWHM Gaussian filter, leading to a voxel size of 1.65 × 1.65 × 1.65 mm^3^. The standard correction methods available on the clinical scanner were employed to address random coincidences, scatter, attenuation, and radioactive decay. For image reconstruction, a point spread function (PSF) + time-of-flight (TOF) algorithm was utilized with 4 iterations and 5 subsets.

### Reference imaged derived input functions

A reference IDIF was generated from a manually selected volume of interest in the descending aorta (DA), and is denoted here by $IDIF_{DA}$ (Image-Derived Input Function from DA). Specifically, mean activity in each time frame was obtained from a cylindrical volume of interest of diameter 10 mm and a length of 10 mm placed over the lumen of the DA. DA was selected based on recent research that compared five different blood pools [2]. This choice was favored due to its minimal susceptibility to cardiac and respiratory motion, along with its larger diameter that mitigates partial volume effects [2]. Despite not using motion correction in our study, we thoroughly assessed descending aorta volume of interest visually, particularly in later frames for accurate tail delineation and manual positioning of ROI.

### Framework for $IDIF_{Auto}$ extraction

**Fig. 1:**
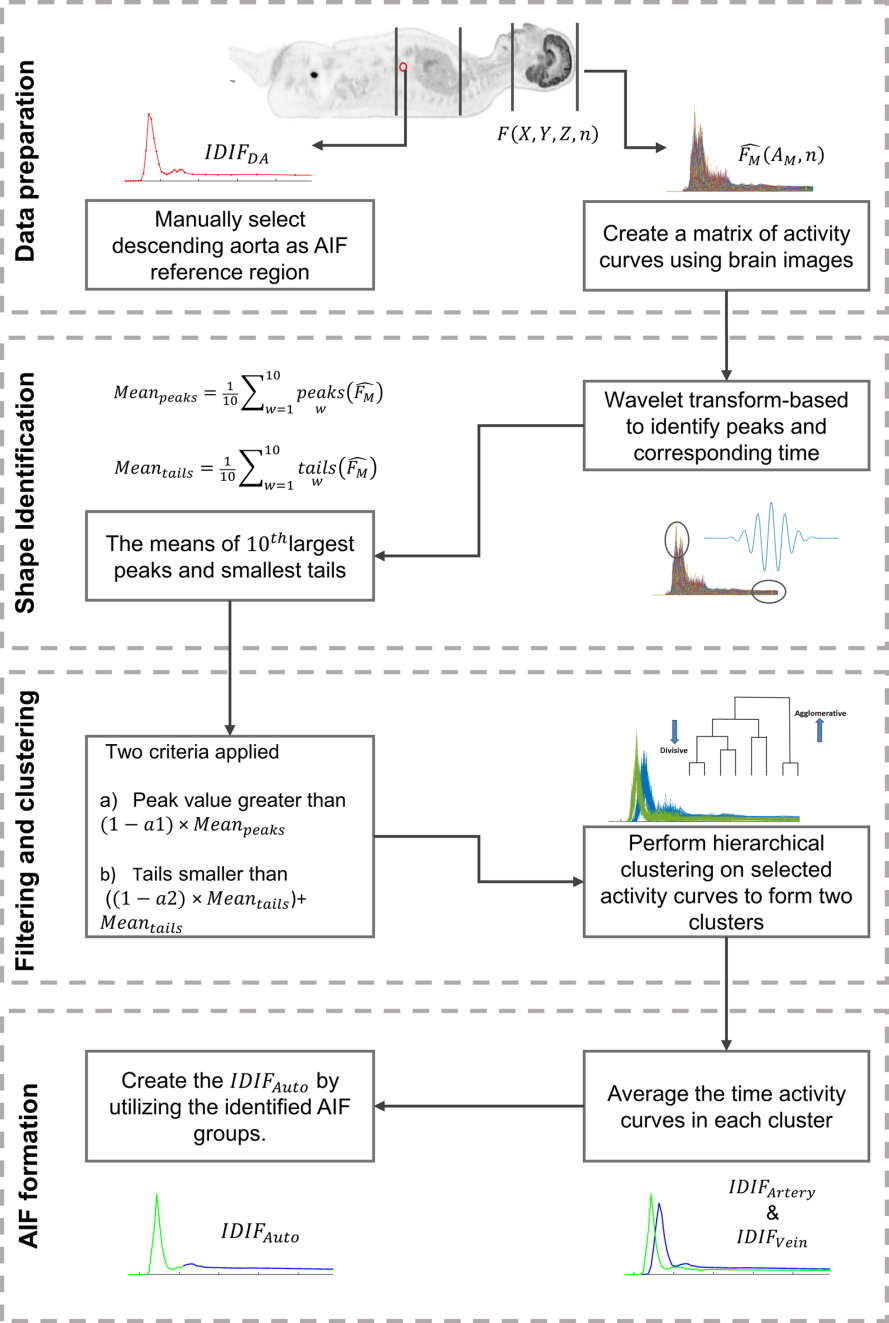
Illustration of the automated framework for extracting the Image-Derived Input Function (IDIF) from brain images

Automated brain IDIF ($IDIF_{Auto}$) extraction used a voxel-based search to identify AIF-like shapes from image time activity curves, taking into consideration both the peak and tail of each curve. This was achieved through the following steps (see Fig. 1):

1. *Data preparation* To create a matrix of activity curves using brain images, a 2D matrix $\widehat{{F_{M} }}\left( {A_{M} ,n} \right)$ was formed from the 4D image $F\left( {X,Y,Z,n} \right)$, where $\hat{F}$ represents the 2D transformed activity curve matrix from the 4D image. The matrix $\hat{F}$ was constructed by selecting all brain voxels and arranging their activity values $A$ into $\hat{F}$ (location versus time), where $M$ denotes the total number of voxels encompassed within the field of view ($X \times Y \times Z$) and $n$ is the number of time frames. Brain voxels with time activity curves were chosen by applying a fixed threshold of one to the averaged image over the time series. This ensures the selection of non-zero voxels containing brain in the images.
  The brain region were chosen to encompass a length coverage of 20 cm of head. This range matches the coverage of the latest clinical PET machines, which typically have a length of 15 to 26 cm [37].
2. *Shape identification* A wavelet transform-based method [38] was used to identify peaks and their corresponding times in each activity curve.
  The tail activity value for each activity curve was considered based on the last two timeframes ($55 - 65\;{\text{min}}$). Subsequently, we computed the averages of the top 10 peaks (referred to as “$Mean_{peak}$“) and the least 10 tail time activity curves (referred to as “$Mean_{tail}$“). This approach of considering the 10 largest peaks and smallest tails was adopted to minimize potential noise influences, a more robust alternative to evaluating solely the single largest peak or smallest tail.
3. Filtering and clustering: We employed two criteria to filter non-AIF shaped activity curves. The first criterion involved selecting curves with peaks greater than $\left( {1 - a1} \right)$ $\times$ $Mean_{peak}$, where 9 specific thresholds ($a1 = 0.1\; to$ $0.9$, $steps = 0.1$) were used to filter out curves originating from tissue and those with high partial volume effects because these were expected to have a lower peak. The second criterion accepted curves with tails smaller than $\left( {\left( {1 - a2} \right) \times Mean_{tail} } \right) + Mean_{tail}$, where 9 specific thresholds (ranging from $a2 = 0.1 to$ $0.9$, $steps = 0.1$) were investigated to filter out curves with high activity values in the tail of the time activity curve, assumed to reflect tissue ^18^F-FDG uptake. Following the filtering stage, the chosen activity curves were classified into two groups through hierarchical clustering [39].
  The processes of shape identification, filtering, and clustering were implemented in MATLAB® R2021b (MathWorks, Natick, MA). For shape identification, the continuous wavelet transform method was employed, utilizing the built-in function ‘CWT’ with setting of using the “Morlet” wavelet function with scales ranging from 1 to 100 and a threshold of 0.5 for significant coefficients (these settings are based on the requirements of the CWT function in MATLAB® R2021b). For clustering, we employed hierarchical clustering, an unsupervised machine learning approach. We utilized the ‘ward’ linkage method to group the selected activity curves and divided the dendrogram into two clusters using the ‘maxclust’ option.
4. *IDIF formation* Two clusters were identified and averaged. The averaged curves had different shapes and peak latencies, as expected from venous and arterial time activity curves and were labeled as $IDIF_{Artery}$ and $IDIF_{Vein}$, representing the image-derived arterial and venous input functions, respectively.

To compare these clustered curves with the $IDIF_{DA}$, the 65-min imaging window was divided into seven time periods ($T_{p}$, $p$ ranging from 1 to 7): $T_{1}$ was selected within the timeframe of 0 min to 20 s after the time of identified peak to ensure that it captures the highest point of the curve in the initial period, $T_{2} = T_{1} + 10\;{\text{min}}$, $T_{3} = T_{2} + 10\;{\text{min}}$, $T_{4} = T_{3} + 10\;{\text{min}}$, $T_{5} = T_{4} + 10\;{\text{min}}$, $T_{6} = T_{5} + 10\;{\text{min}}$ and $T_{7}$ extends from the end of $T_{6}$ to the end of the 65-min acquisition period. The area under the curve ($AUC$) for each time period ($AUC_{{T_{p} }}^{Artery}$ and $AUC_{{T_{p} }}^{Vein}$) was calculated, and compared with the $AUC$ for the $IDIF_{DA}$ ($AUC_{{T_{p} }}^{DA}$, $p$ ranging from 1 to 7). The $IDIF_{Auto}$ was determined by selecting the combination of $IDIF_{Artery}$ and $IDIF_{Vein}$ that had the lowest $AUC$ error across $T_{1}$ to $T_{7}$.

### $IDIF_{Auto}$evaluation

The goodness of $IDIF_{Auto}$ estimation was assessed using the $AUC$ and Normalised Root Mean Square Error ($NRMSE$) compared to $IDIF_{DA}$. The $AUC$ error was calculated using:

1 $$AUC_{error} = \frac{{AUC - \widehat{{AUC^{ } }}}}{{\widehat{{AUC^{ } }}}} \times 100,$$

where $AUC_{error}$ is the percentage error, $AUC$ is the AUC for the estimated IDIF and $\widehat{{AUC^{ } }}{ }$ is the AUC for the $IDIF_{DA}$. The $NRMSE$ was calculated as:

2 $$NRMSE = \sqrt {\frac{1}{T}\mathop \sum \limits_{t = 1}^{T} \frac{{\left( {f_{t}^{ } - \widehat{{f_{t}^{ } }}} \right)^{2} }}{{\widehat{{f_{t}^{ } }}^{2} }}} ,$$

where ${f}_{t}$ denotes the estimated IDIF at the *t*^*th*^ time point, $\widehat{{{f}_{t}}}$ is the corresponding value for the ${IDIF}_{DA}$, and $T$ is the total number of timepoints in the IDIF.

### Exploring patient information variations and evaluating algorithm performance by weight

To investigate potential variations in patient information within the model adjustment and validation groups, we analysed age and weight data from Additional file 1: Table S1 and reported the corresponding results of statistical tests. Moreover, irrespective of adjustment and validation groups, we evaluated the algorithm’s performance based on patient weight by organizing the dataset into three weight groups: Group 1, comprising four subjects with the highest weights (97 ± 22 kg); Group 2, including four subjects with medium weights (74 ± 5 kg); and Group 3, encompassing four subjects with the lowest weights (57 ± 6 kg). Mean and standard deviation values of $AUC_{error}$ and $NRMSE$ were presented, accompanied by the results of statistical tests.

### The impact of different PSF Settings (FWHM of the Gaussian kernel) on the accuracy of $IDIF_{Auto}$

To evaluate the performance of our framework at different image resolutions, we applied additional Gaussian blurring using 3D kernels with FWHMs of 1mm, 2mm, 3mm, 4mm, and 5mm to the images. Following additional blurring, the resulting image resolutions were FWHM of 3.95mm, 4.31mm, 4.86mm, 5.53mm, and 6.29mm, calculated using $FWHM = \sqrt {\left( {FWHM_{1} } \right)^{2} + \left( {FWHM_{2} } \right)^{2} + \left( {FWHM_{3} } \right)^{2} }$. The native in-plane spatial resolution, denoted as $FWHM_{1}$, was 3.27mm [36]. Additional smoothing with a Gaussian filter of FWHM 2mm ($FWHM_{2}$) was applied to the original PET data during reconstruction. $FWHM_{3}$ represents further Gaussian blurring applied to assess different PSF settings. The $AUC_{error}$ and $NRMSE$ at each of these resolutions was calculated as above. We also evaluated the average number of voxels identified as veins and arteries at each new FWHM value.

### Pixelwise kinetic modelling

Time activity curves were fitted using the irreversible two tissue compartment model (2TCM):

3 $$C_{T} \left( t \right) = \left( {1 - v_{b} } \right)\left( {\left( {\frac{{K_{1} k_{2} }}{{k_{2} + k_{3} }}e^{{ - \left( {k_{2} + k_{3} } \right)t}} + \frac{{K_{1} k_{3} }}{{k_{2} + k_{3} }}} \right) \otimes C_{p} \left( t \right)} \right) + v_{b} C_{b} \left( t \right),$$

where $C_{T} \left( t \right)$ represents the measured total tracer concentration in tissue, $C_{p} \left( t \right)$ and $C_{b} \left( t \right)$ represent the concentration of tracer in plasma and blood, $t$ (in min) is a point in time, $v_{b}$ represents the fraction of volume occupied by the tracer in the blood pool, while the symbol ⨂ denotes the convolution operation. Parameters $K_{1}$ (${\text{ml}}/cm^{3} /{\text{min}}$), $k_{2}$ ($1/{\text{min}}$), and $k_{3}$($1/{\text{min}}$) are the kinetic parameter respectively representing tracer influx and efflux rates between blood and tissue and the rate of phosphorylation ^18^F-FDG [4–6]. Kinetic parameters were generated using both $IDIF_{DA}$ and $IDIF_{Auto}$ for $C_{b} \left( t \right)$. Equation (3) was fitted using the nonlinear least squares method, utilizing lsqcurvefit, a built-in function available in MATLAB® 2021. For optimization, the Levenberg–Marquardt (LM) algorithm [40] was employed. The initial values for fitting $K_{1}$, $k_{2}$, $k_{3}$, and $v_{b}$ were set to 0.01. The lower bounds for all parameters were set to zero, while the upper bounds were set to one. Spatially resolved parametric maps for $K_{1}$, $k_{2}$, and $k_{3}$ were generated for each brain. The net influx rate constant, representing the overall rate of tissue tracer uptake:

$${ }K_{i} = \frac{{K_{1} k_{3} }}{{\left( {k_{2} + k_{3} { }} \right)}}{ }\left( {{\text{ml}}/\text{cm}^{3} /{\text{min}}} \right)$$

was computed directly from the kinetic parameters.

No extra smoothing, filtering, or manual outlier adjustments were implemented to handle noisy data.

### Patlak analysis

We applied the Patlak linear graphical plot method to the 40–65 min data from each brain time activity curve, to ensure that pseudo-equilibrium was achieved [2], and $K_{i}$ maps were generated using $IDIF_{DA}$ and $IDIF_{Auto}$ using the function lsqlin, a linear least-squares fitting algorithm implemented in MATLAB®.

### Statistical analysis

Coefficient of determination ($R^{2}$) and linear regression analysis were performed to assess the correlation between the parametric maps estimated using $IDIF_{DA}$ and $IDIF_{Auto}$. To compare the estimated parameters derived from different input functions, we presented the mean, standard deviation, and error, and also conducted a paired Student’s t-test. A significance level of 0.05 was used to determine statistical significance. The verification of normality assumptions was conducted through the Shapiro-Wilks test [41] with a significance level set at *p* < 0.05.

## Results

### Threshold level selection for clustered time activity curves

**Fig. 2:**
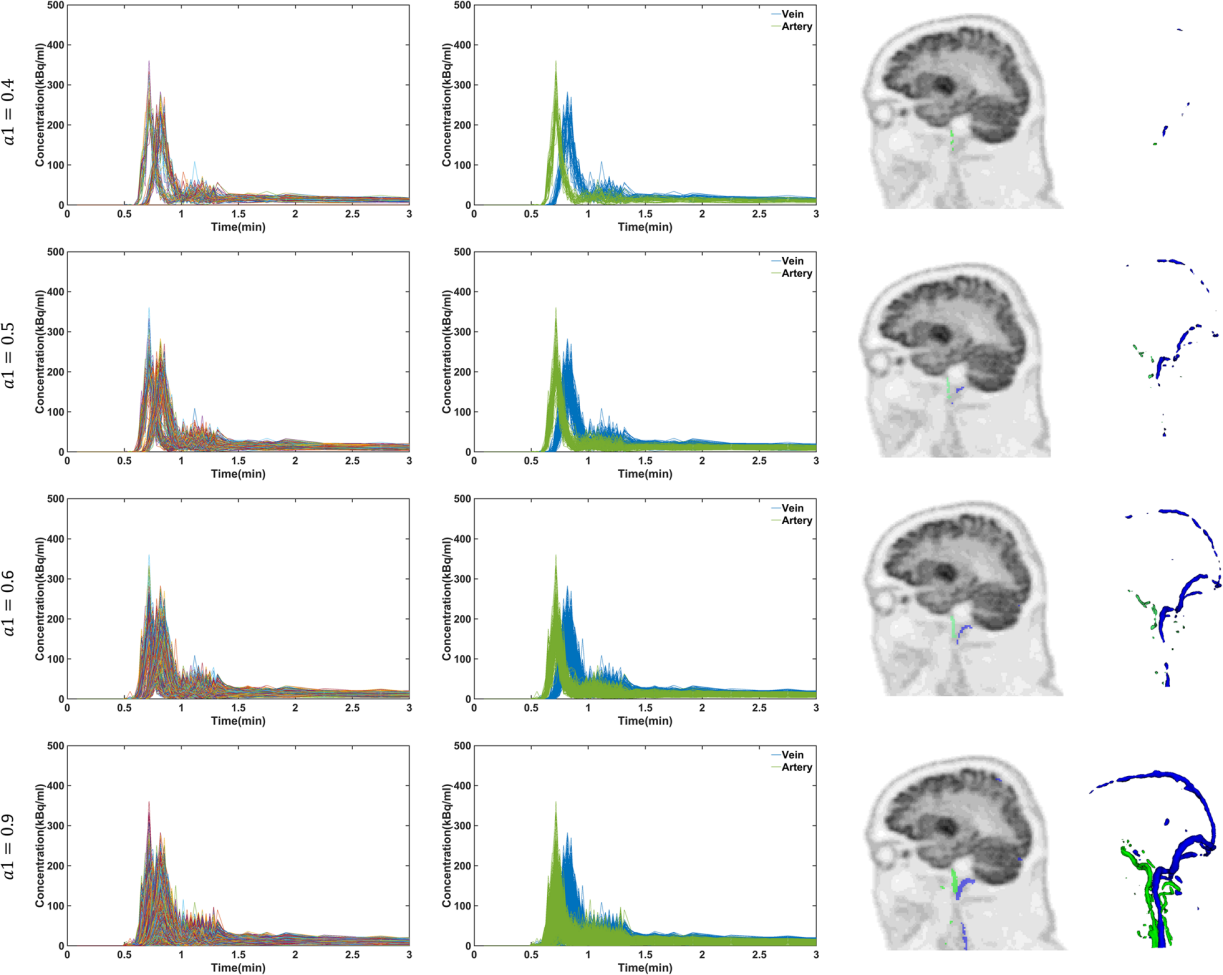
First column shows of the identified voxels in the brain associated with activity curves for participant 6 at four different threshold levels ($a1=0.4, 0.5, 0.6$ and $0.9$ when $a2=0.9$). Second column shows the clustered activity curves with early and late peaks, respectively, labelled as arteries (green lines) and veins (blue lines). The maps in third and fourth columns show the location of the curves back onto the anatomical regions from which they were extracted, clearly showing the location of large arteries (green pixels) and large veins (blue pixels)

Figure 2 shows the identified voxels in the brain associated with activity curves for participant 6. The impact of setting $a1 =$ 0.4, 0.5, 0.6 and $0.9$ when $a2 = 0.9$ on the extracted data are shown. Two clusters can be discerned for each of the two threshold levels, and a difference in time to peak between the activity curves in these clusters is apparent. To differentiate between the two clusters, the activity curves with early peaks were labelled as arteries, while those with late peaks were labelled as veins, as shown in Fig. 2. The voxel locations corresponding to each of the curves, corresponded to the expected locations of arteries and veins. These findings were consistent across all participants.

Upon visual inspection, voxels with the largest peaks were consistently linked to the lowest tails, primarily originating from large artery vessels.

Additional file 1: Table S2 compares the averaged clustered IDIFs with the reference $$IDIF_{DA}$$ and shows the $$AUC_{error}$$ for each cluster for different threshold levels ($$a1 = 0.1 to 0.9).$$ Our results showed that the $$a2$$ threshold level had a minor impact on the extracted IDIFs; we chose $$a2 = 0.9$$ (only activity curves with tails smaller than $$\left( {\left( {1 - a2} \right) \times Mean_{tail} } \right) + Mean_{tail}$$) to minimise the likelihood of including high uptake tissue activity curves. We found that the average $$AUC_{error}$$ for $$IDIF_{Artery}$$ was about four times larger than for the $$IDIF_{Vein}$$ irrespective of the threshold level and participant. Additionally, the average $$AUC_{errors}$$ for $$IDIF_{Vein}$$ were − 1.98 ± 7.37%, − 2.55 ± 6.34% and − 3.80 ± 7.01% at the respective 0.4, 0.5, and 0.6 threshold levels. For the same threshold levels, the $$AUC_{errors}$$ of $$IDIF_{Artery}$$ were − 16.37 ± 7.05%, − 19.61 ± 4.17%, and − 20.44 ± 4.01%. These three threshold levels were identified as optimal among the six participants, as the $$AUC_{errors}$$ for $$IDIF_{Vein}$$ were found to be the lowest for each specific threshold level, with two participants exhibiting the minimum error for each level. As such, input functions were created by averaging across these three levels, yielding $$IDIF_{Vein}^{0.4 - 0.6}$$ and $$IDIF_{Artery}^{0.4 - 0.6}$$. The $$AUC_{error}$$ for $$IDIF_{Vein}^{0.4 - 0.6}$$ and $$IDIF_{Artery}^{0.4 - 0.6}$$ were found to be − 2.97 ± 4.44% and − 18.76 ± 6.36%, respectively.

### $IDIF_{Artery}$and $IDIF_{Vein}$ compared to $IDIF_{DA}$ and formation of $IDIF_{Auto}$

Additional file 1: Table S3 presents the $AUC_{errors}$ in $T_{1}$ to $T_{7}$, comparing $IDIF_{Vein}^{0.4 - 0.6}$ and $IDIF_{Artery}^{0.4 - 0.6}$ with the reference $IDIF_{DA}$. The findings highlight that during $T_{1}$, which included the initial input function peak, the averaged $AUC_{error}$ across participants for $IDIF_{Artery}^{0.4 - 0.6}$ (− 1.36%) was lower than for $IDIF_{Vein}^{0.4 - 0.6}$(15.84%). However, for all other time periods, the $AUC_{error}$ for $IDIF_{Vein}^{0.4 - 0.6}$ was lower than that for $IDIF_{Artery}^{0.4 - 0.6}$.

Based on our findings, we observed that the optimal approach is to combine the initial peak of $IDIF_{Artery}^{0.4 - 0.6}$ with the remaining portion after the first peak from $IDIF_{Vein}^{0.4 - 0.6}$ to obtain $IDIF_{Auto}$. To achieve this, we interpolated the AIF shape to a lower time frame period of 2 s. To minimize any discontinuity and step-like shape in the final AIF, we averaged values in $IDIF_{Artery}^{0.4 - 0.6}$ and $IDIF_{Vein}^{0.4 - 0.6}$ for two time points (4s) before and after the concatenation point, replacing them with the actual values to obtain $IDIF_{Auto}$. We then interpolated back to the original 62 frames in this study. Table 1 reports the delay in seconds between IDIFs derived from the descending aorta and brain arteries and veins for each subject (P1 to P6). No significant difference in mean time to peak was observed between the descending aorta and brain arteries (delay = 0.66 ± 1.03 s, paired t-test, *P* = 0.17), with the mean delay with respect to brain veins being 6.00 ± 1.26 s and 6.66 ± 1.63 s respectively. A maximum delay between the descending aorta and brain vein of 8 s was observed for P1, P3 and P4, while a minimum delay of 4 s occurred for P2. No significant delay was found between the vein IDIF and tissue time activity curves (delay = 0.33 ± 0.81 s, paired t-test, P = 0.36). In the formation of $IDIF_{Auto}$, we first calculated the difference in time to peak between the arterial and venous IDIFs and shifted the time points of the venous curve to align the peaks of the arterial and venous IDIFs.

**Table 1:** Comparison of delay in seconds between IDIFs derived from the descending aorta, brain arteries, brain veins, and tissue time activity curve (TTAC) for each subject in the adjustment group (P1 to P6)

Delay (seconds)	Artery versus DA	Vein versus DA	TTAC versus DA	Vein versus artery	TTAC versus vein
P1	0	8	8	8	0
P2	0	4	6	4	2
P3	2	8	8	6	0
P4	2	8	8	6	0
P5	0	6	6	6	0
P6	0	6	6	6	0
Mean ± SD	0.66 ± 1.03	6.66 ± 1.63	7 ± 1.09	6 ± 1.26	0.33 ± 0.81

### $IDIF_{Auto}$for the adjustment cohort (n = 6)

**Fig. 3:**
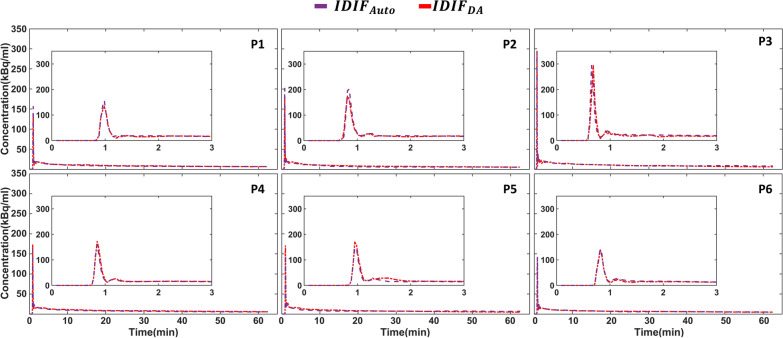
Displays a comparison between the arterial input function automatically extracted from brain images (${IDIF}_{Auto}$) and the descending aorta IDIF (${IDIF}_{DA}$) for the six subjects from the adjustment cohort (P1–P6). The ${IDIF}_{Auto}$ is represented by the purple dashed line, and the ${IDIF}_{DA}$ is shown as the red dashed-dotted line

Figure 3 displays the $IDIF_{Auto}$ for the six subjects from the adjustment cohort. The automatically extracted IDIFs for all six accurately captured the shape of $IDIF_{DA}$. Table 2 summarises participants’ $AUC_{error}$ and $NRMSE$ values. The average $AUC_{error}$ was − 4.31% and the mean $NRMSE$ was 0.21.

**Table 2:** Comparison of $AUC_{error}$ and $NRMSE$ between the automatically extracted arterial input function from brain images ($IDIF_{Auto}$) and the descending aorta IDIF ($IDIF_{DA}$) for six subjects in the adjustment cohort (P1-P6)

	${AUC}_{error}$ (%)	$NRMSE$
P1	− 5.87	0.15
P2	− 10.98	0.20
P3	4.54	0.45
P4	− 11.89	0.18
P5	− 2.70	0.18
P6	1.02	0.11
Mean ± SD	− 4.31 ± 6.54	0.21 ± 0.12

### $IDIF_{Auto}$for the validation cohort (n = 6)

**Fig. 4:**
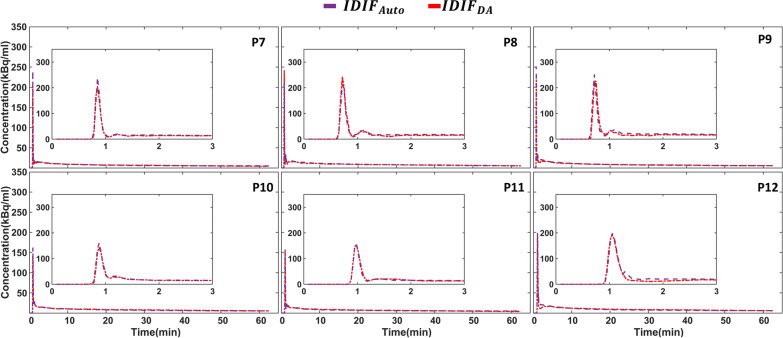
Displays a comparison between the arterial input function automatically extracted from brain images (${IDIF}_{Auto}$) and the descending aorta IDIF (${IDIF}_{DA}$) for the six subjects from the validation cohort (P7–P12). The ${IDIF}_{Auto}$ is represented by the purple dashed line, and the ${IDIF}_{DA}$ is shown as the red dashed-dotted line

To validate $IDIF_{Auto}$, the approach was applied to a validation group of six additional subjects (Fig. 4). Automatically extracted IDIFs for all subjects in the validation group were consistent with individuals’ $IDIF_{DA}$ on visual inspection. The performance metrics, including the mean $AUC_{error}$ and $NRMSE$ values, showed slightly better results for the validation group compared to the adjustment cohort, as depicted in Table 3. Specifically, the mean $AUC_{error}$ and $NRMSE$ values in the validation group were − 1.59% and 0.17, respectively, whereas in the adjustment group, they were 4.31% and 0.21.

**Table 3:** Comparison of $AUC_{error}$ and $NRMSE$ between the automatically extracted arterial input function from brain images ($IDIF_{Auto}$) and the descending aorta IDIF ($IDIF_{DA}$) for six subjects in the adjustment cohort (P7–P12)

	${AUC}_{error}$ (%)	$NRMSE$
P7	0.42	0.17
P8	0.81	0.18
P9	1.49	0.32
P10	− 5.84	0.14
P11	− 2.69	0.10
P12	− 3.71	0.13
Mean ± SD	− 1.59 ± 2.93	0.17 ± 0.07

### Analysing subject disparities in adjustment and validation groups, and evaluating algorithm performance by patient weight

In exploring potential factors influencing cohort performance metrics, it is noteworthy that no significant age difference existed between the adjustment group (61 ± 19 years) and the validation group (62 ± 18 years), as determined by a paired Student’s t-test (*P* = 0.9). Similarly, no significant weight difference was observed (87 ± 25 kg and 66 ± 9 kg, respectively; paired t-test, *P* = 0.14).

The algorithm’s performance was similar in the groups defined according to patient weight with no significant differences in $AUC_{error}$ being observed in comparisons between Group 1 vs Group 2, Group 3 vs Group 2, and Group 1 vs Group 3 (*P* = 0.39, 0.5, and 0.97, respectively; Group 1: 3.7 ± 6.49, Group 2: − 1.14 ± 2.4, and Group 3: − 3.9 ± 6.19). Similarly, there were no significant differences in $NRMSE$ (*P* = 0.24, 0.054, and 0.66, respectively; Group 1: 0.24 ± 0.13, Group 2: 0.13 ± 0.03, and Group 3: 0.2 ± 0.08) between the three groups, indicating that body weight had no observable impact on cohort performance metrics.

### The impact of image resolution on the accuracy of $IDIF_{Auto}$

**Fig. 5:**
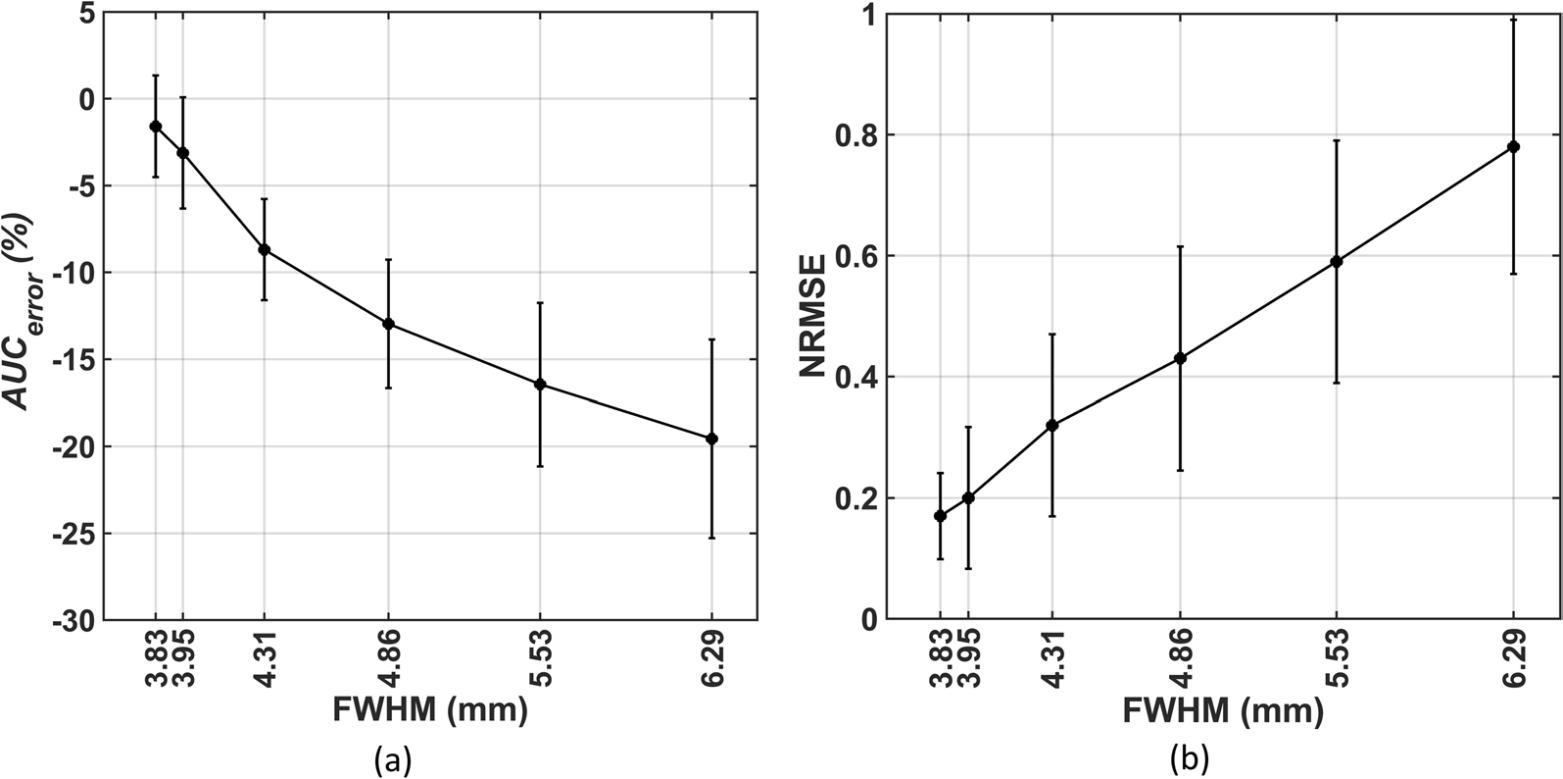
Illustration of the impact of different PSF Settings (FWHM of the 3D Gaussian kernel) on the accuracy of ${IDIF}_{Auto}$ in the validation group (n = 6)

Figure 5 shows that the mean and SD of the $AUC_{error}$ and $NRMSE$ increases with FWHM. Without additional blurring, $IDIF_{Auto}$ underestimated $IDIF_{DA}$ with a mean AUC and $NRMSE$ of 1.59% and 0.17. At a FWHM of 3.95mm, mean $AUC_{error}$ and $NRMSE$ were − 5% and 0.20 in the validation group. The highest $AUC_{error}$, was around 20% underestimation, at a resolution of 6.29mm FWHM.

**Fig. 6:**
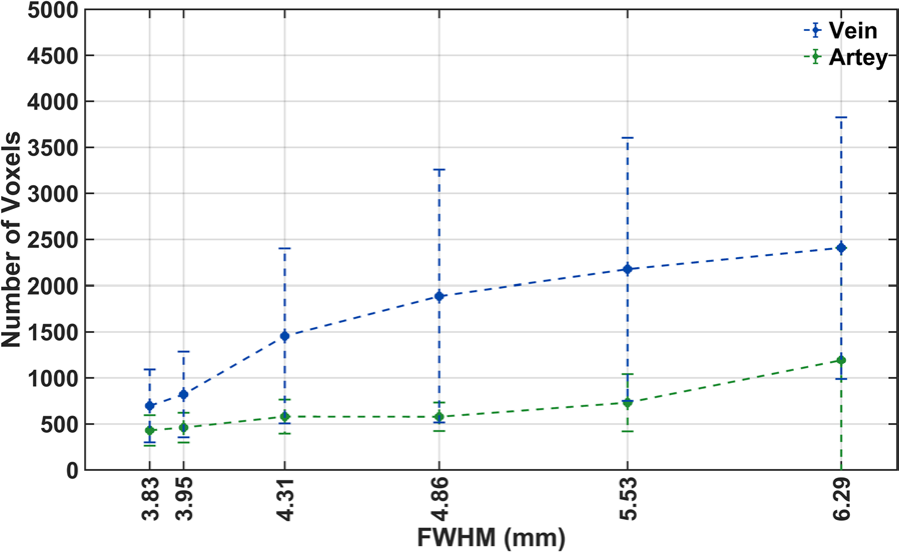
Illustration of the impact of different PSF Settings (FWHM of the 3D Gaussian kernel) on the number of voxels identified as vein and artery in the validation group (n = 6)

Figure 6 displays the average number of voxels identified as veins and arteries at threshold levels of 0.4, 0.5, and 0.6 for different FWHM values in the validation group. At a FWHM setting of 3.83mm, mean and standard deviation of the number of voxels identified as arteries and veins were 431 ± 165 and 698 ± 395 respectively. At all FWHMs, more voxels were identified as venous than arterial and the difference between venous and arterial voxel number increased with FWHM in all participants.

### Parametric mapping and Patlak analysis

**Fig. 7:**
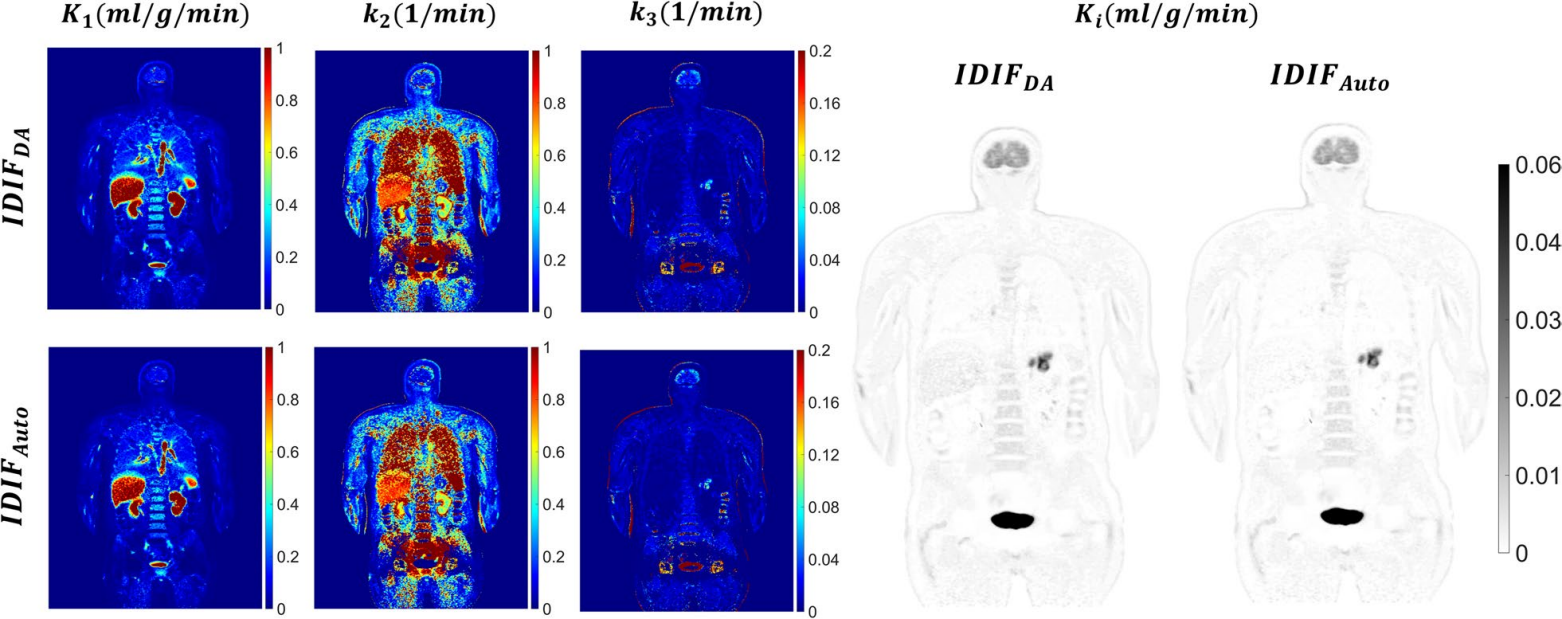
Coronal views of ^18^F-FDG parametric images for a lymphoma patient (subject 6). The figure shows parameter maps of $K_{1}$ (${\text{ml}}/{\text{cm}}^{3} /{\text{min}}$), $k_{2}$ ($1/{\text{min}}$), $k_{3}$ ($1/{\text{min}}$), and $K_{i}$ (${\text{ml}}/{\text{cm}}^{3} /{\text{min}}$), obtained using AIF extracted from the descending aorta ($IDIF_{DA}$) and brain with an automated framework ($IDIF_{Auto}$). The AIF errors between the two methods were $AUC_{error}$=1.02% and *NRMSE* = 0.11

**Fig. 8:**
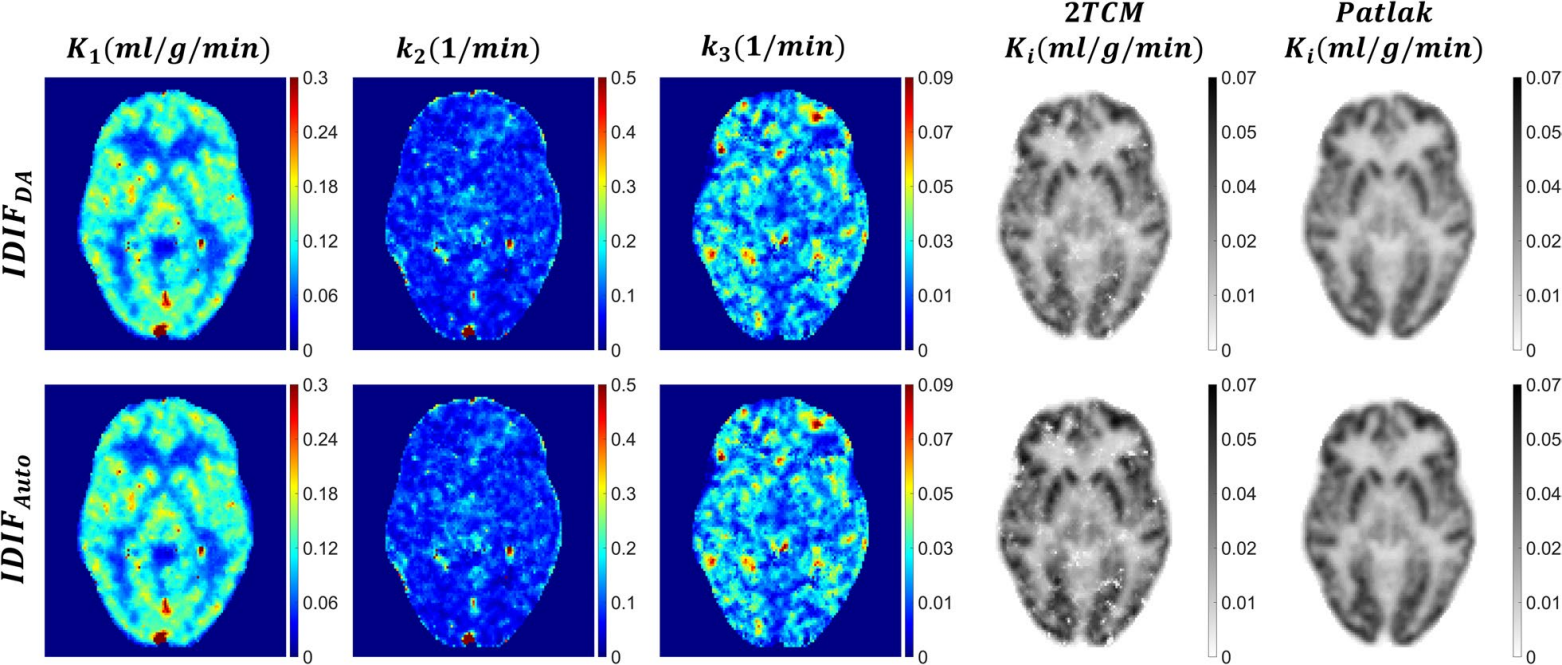
Illustration of axial parameter maps of $K_{1}$ (${\text{ml}}/cm^{3} /{\text{min}}$), $k_{2}$ ($1/min$), $k_{3}$ ($1/min$), and $K_{i}$ (${\text{ml}}/cm^{3} /{\text{min}}$) from subject 10, showing a comparison between AIF extracted from the descending aorta ($IDIF_{DA}$) and brain with an automated framework ($IDIF_{Auto}$). The AIF errors between the two methods were $AUC_{error}$ = − 5.84% and *NRMSE* = 0.14

The parametric maps in Figs. 7 and 8 were generated using a two tissue compartment model and provide a comparison of $IDIF_{Auto}$ and $IDIF_{DA}$ in two representative oncological subjects. Visual inspection of the $K_{i}$ maps obtained using $IDIF_{Auto}$ and $IDIF_{DA}$ did not detect qualitative differences. Minor qualitative differences were evident for $K_{1}$, $k_{2}$ and $k_{3}$ maps in some subjects in keeping with the sensitivity of these parameters to slight changes in AIF shape.

**Fig. 9:**
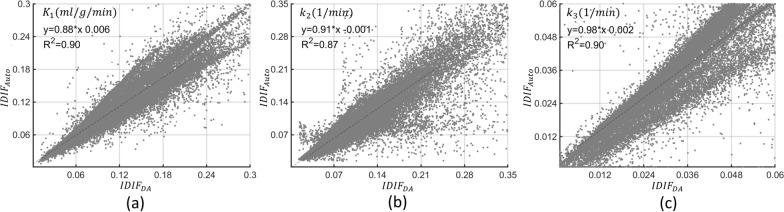
Voxel-wise scatter plot for ${K}_{1}$ (**a**), ${k}_{2}$ (**b**), and ${k}_{3}$ (**c**) for the six subjects from the validation cohort (P7–P12) showing the coefficient of determination (${R}^{2}$) and linear regression analysis for the correlation between the parametric maps estimated using the descending aorta IDIF (${IDIF}_{DA}$) and the automatically extracted image-derived input function from brain images (${IDIF}_{Auto}$)

**Fig. 10:**
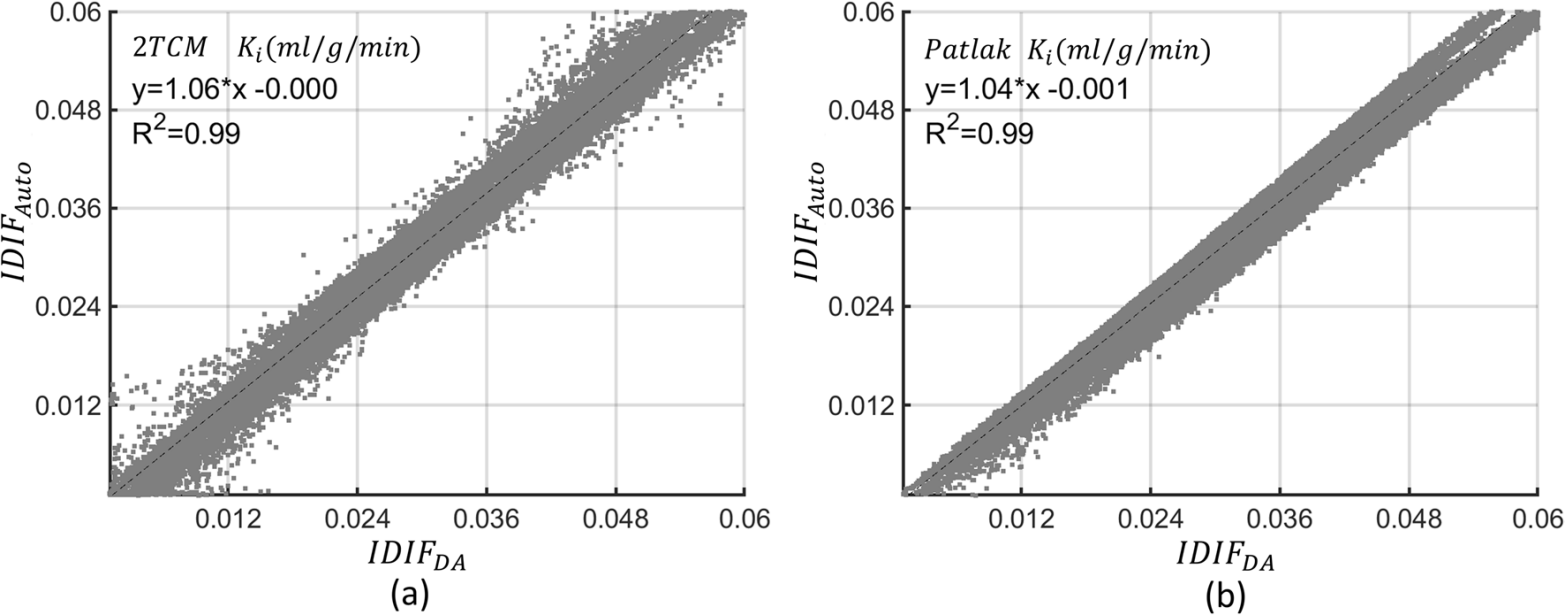
Voxel-wise scatter plot for $2TCM {K}_{i}$ (**a**) and $Patlak {K}_{i}$ (**b**) for the six subjects from the validation cohort (P7–P12) showing the coefficient of determination (${R}^{2}$) and linear regression analysis for the correlation between the parametric maps estimated using the descending aorta IDIF (${IDIF}_{DA}$) and the automatically extracted image-derived input function from brain images (${IDIF}_{Auto}$)

When we compared scatter plots (Figs. 9 and 10) of kinetic parameters at each brain voxel, estimated using each IDIF, $R^{2}$ exceeded 0.87 for $K_{1}$, $k_{2}$ and $k_{3}$, and was 0.99 for $K_{i}$ when either AIF estimation method was used. Individual rate constant estimates ($K_{1}$, $k_{2}$, $k_{3}$) display higher variability than $K_{i}$ (Additional file 2: Fig. 1 and Additional file 3: Fig. 2), with the $R^{2}$ differing by less than 1 (Fig. 9).

The regression line slopes for $K_{i}$ in both the 2TCM and Patlak analyses were approximately 1.06 and 1.04, slightly exceeding one (Fig. 10). Conversely, the slopes for $K_{1}$, $k_{2}$ and $k_{3}$ were 0.88, 0.91, and 0.98, slightly falling below one (Fig. 9).

The results of kinetic parameter estimation in gray and white matter using $IDIF_{Auto}$ and $IDIF_{DA}$ are presented in Additional file 1: Table 4. $K_{1}$, $k_{2}$, and $k_{3}$ estimates for gray matter using $IDIF_{DA}$ were 0.173 ± 0.039 ${\text{ml}}/{\text{cm}}^{3} /{\text{min}}$, 0.137 ± 0.054, and 0.053 ± 0.015$1/{\text{min}}$, respectively. Corresponding estimates for white matter were 0.061 ± 0.021 ${\text{ml}}/{\text{cm}}^{3} /{\text{min}}$, 0.094 ± 0.049, and 0.025 ± 0.015 $1/min$. These values are consistent with previously reported ranges [42, 43].

Our analysis of the $K_{1}$, $k_{2}$ and $k_{3}$ values estimated in gray and white matter using both input functions revealed no statistically significant differences except for $k_{2}$ values for white matter ($K_{1}$: *p* = 0.77 (GM), *p* = 0.40 (WM); k2: *p* = 0.20 (GM), *p* = 0.0064 (WM); $k_{3}$: *p* = 0.65 (GM), *p* = 0.26 (WM); paired t-test). The mean $K_{i}$ values for gray and white matter estimated using 2TCM with $IDIF_{DA}$ were 0.048 ± 0.006 and 0.011 ± 0.004 ${\text{ml}}/{\text{cm}}^{3} /{\text{min}}$, respectively, in close agreement with those obtained using the $IDIF_{Auto}$ (correspondingly 0.047 ± 0.007 and 0.011 ± 0.004 $\left( {ml/cm^{3} /min} \right);p > 0.05)$. The mean percentage errors for $K_{1}$, $k_{2}$ and $k_{3}$ in gray matter were − 0.7 ± 12.3%, − 3.5 ± 6.3%, and 4.9 ± 15.4%, respectively and, correspondingly, for white matter were − 2.9 ± 12.6%, − 12.4 ± 5.8%, and − 3.5 ± 19.3%. 2TCM and Patlak estimates of $K_{i}$ yielded errors for gray matter of 2.8 ± 1.7% and 1.9 ± 4.5%, respectively, while for white matter, the corresponding errors were − 0.3% ± 7.9% and 3.7% ± 5.5%. These results suggest that $K_{i}$ was less influenced by input function shape and was primarily determined by $AUC_{{{\text{error}}}}$, whereas individual parameters were highly sensitive to input function shape.

Additional file 4: Fig. 3 depicts mean values for both GM and WM across $K_{1}$, $k_{2}$, $k_{3}$, $2TCM{ }K_{i}$, and $Patlak{ }K_{i}$ for the six subjects in the validation cohort (values in Additional file 1: Table 4). The plots depict $R^{2}$, the slope and the 95% confidence interval, highlighting the variability in $K_{1}$, $k_{2}$, and $k_{3}$ estimates. Of note, the 95% confidence intervals for the slope of the regression line for $K_{1}$, $k_{2}$, and $k_{3}$ were 0.77 to 1.117, 0.86 to 1.103, and 0.76 to 1.103, respectively. For $2TCM K_{i}$ and $Patlak{ }K_{i}$, the 95% confidence intervals for the slope of the regression line were 0.99 to 1.106 and 0.95 to 1.059. Similarly, the 95% confidence intervals for the $R^{2}$ values ranged from 0.88 to 0.99 for $K_{1}$, $k_{2}$, and $k_{3}$, and from 0.995 to 0.999 for $2TCM K_{i}$ and $Patlak{ }K_{i}$.

## Discussion

Our study presents a simple automated framework for extracting the IDIF from ^18^F-FDG-PET brain images. A wavelet transform-based method was used to identify the peak of each time activity curve and hierarchical cluster analysis was then employed to separate arterial from venous curves. This yielded an estimated IDIF (${IDIF}_{Auto}$) that combined components of the arterial and venous AIF and had a temporal profile that was very close to that of a reference aortic IDIF (${IDIF}_{DA}$). Our framework eliminates corrections for partial volume effect and simplifies parametric brain PET when limited field-of-view scanners are available.

We utilized the IDIF obtained from the descending aorta (${IDIF}_{DA}$) as the reference arterial input function, as previous studies have validated its accuracy against arterial blood sampling, demonstrating AUC correlations of 0.99 [44] and 0.91 [45]. However, arterial blood sampling yields an AIF that differs from the AIF in the descending aorta due to dispersion effects. The peak activity in the aorta appears earlier, higher, and narrower than the AIF peak from arterial cannulation [46]. It is anticipated that dispersion-induced changes are negligible between the descending aorta and the arterial vasculature AIF in the brain. Recent research underscores the descending aorta as the optimal option for estimating the reference input function among different cardiac blood pools [2].

We found that utilizing a combination of three thresholds ($a1$: 0.4, 0.5, and 0.6), which are determined based on the maximum identified peak, along with the smallest tail threshold ($a2$: 0.9) as explained in the methodology section, yielded the lowest $AU{C}_{error}$, and allowed the estimation framework to be generalized across subjects.

The automated framework identified the time period consisting peak of the IDIF from brain arteries (${IDIF}_{Artery}$) to be closer to that of the ${IDIF}_{DA}$ than that of veins. This likely reflects delay and dispersion effects as the radiotracer transits through parenchymal vessels. For the remaining duration, the venous IDIF (${IDIF}_{Vein}$) showed better alignment with the ${IDIF}_{DA}$ than the arterial IDIF. The larger diameter of the identified venous structures, as compared to the arteries, is likely to make the former less susceptible to partial volume effects. This concept has been elucidated in previous studies [47–50]. One study [51] utilized Graph-based Mumford-Shah segmentation to extract the internal carotid arteries and venous sinuses, with the aim of estimating non-invasive arterial input function. They found the combined use of internal carotid and venous sinus regions of interest improved the accuracy of estimating the measured plasma input curve compared to using internal carotid ROIs alone. Based on the results of the ${AUC}_{errors}$ for the 7-time period comparison between venous and arterial IDIFs against the reference AIF (refer to Additional file 1: Table 3), we concluded that utilizing a combination of venous and arterial IDIFs would be preferable to minimize the ${AUC}_{errors}$.

In comparing the performance metrics for ${IDIF}_{Auto}$ and ${IDIF}_{DA}$ between the validation and adjustment cohorts (Tables 2 and 3), we observed slightly superior results in the validation group. In the adjustment cohort, an initial exploration involving various threshold levels ($a1=0.1 to 0.9$) took place, leading to the identification of three optimal thresholds. Despite variations in optimal thresholds for individual subjects, the average of these thresholds was chosen as the global threshold for the adjustment cohort. Surprisingly, this global threshold inadvertently contributed to an enhanced algorithm performance observed in the validation cohort. This discrepancy may be attributed to the random data selection process employed for both cohorts.

We also assessed the impact of inaccuracies in IDIF estimation on kinetic parameter estimates. The two tissue compartment model allows estimation of forward and reverse glucose transport (${K}_{1}$ and ${k}_{2}$) and phosphorylation of ^18^F-FDG by hexokinase (${k}_{3}$), which are potentially more sensitive disease biomarkers than ${K}_{i}$ alone [52].

Further interrogation of the results Additional file 1: Table 4, Additional file 2: Fig. 1, and Additional file 3: Fig. 2), variations in individual rate constants (${K}_{1}$, ${k}_{2}$, ${k}_{3}$) compared to ${K}_{i}$ were observed. Voxel-wise Bland–Altman plots (Additional file 2: Fig. 1, and Additional file 3: Fig. 2) illustrate percentage differences in individual rate constants in the validation cohort, revealing more dispersed voxel differences for lower parameter values and higher percentage differences compared to both ${K}_{i}$ estimates from 2TCM and Patlak. Moreover, the ${R}^{2}$ for ${K}_{i}$ exhibits excellent performance, close to one (Fig. 10), while ${R}^{2}$ for individual rate constants estimations were below one (Fig. 9). These discrepancies stem from the unexpected sensitivity of individual rate constants to variations in peak height, shape, and a slight time-shift between ${IDIF}_{Auto}$ and ${IDIF}_{DA}$. The stability of ${K}_{i}$ estimation can be attributed to the cumulative impact of individual rate constants on the ${K}_{i}$ estimation process. This characteristic renders ${K}_{i}$ less vulnerable to errors occurring at early time points and more responsive to the $AUC$ of both ${IDIF}_{Auto}$ and ${IDIF}_{DA}$ [16, 53–55].

The slopes of the regression lines for ${K}_{i}$ in both the 2TCM and Patlak analyses slightly exceeded one (Fig. 10), likely due to underestimation of AUC for ${IDIF}_{Auto}$, consistent with prior research [45]. While ${K}_{i}$ typically correlates with ${K}_{1}$ and ${k}_{3}$ [2], we observed lower slopes for ${K}_{1}$ and ${k}_{3}$ (compared to ${K}_{i}$), potentially due to the spread of data points in the scatter plot (refer to Fig. 9). The disparity between the slopes of the regression lines for ${K}_{i}$ and ${K}_{1}$, particularly in white matter voxels, may be due to the effects of noise on parameter estimation. Furthermore, Additional file 4: Fig. 3 visually illustrates the extent of potential variability in the slopes of the regression lines.

Noisy voxel-wise time activity curves can also introduce errors in the individual rate constants, especially when fitting the compartment model to noisy points in the TAC, resulting in overfitting, particularly in ${k}_{2}$ estimation [56]. To address this issue, strategies such as manual adjustment (excluding specific points) [56], noise-filter application [57], and implementing reasonable parameter limits are employed [58]. In our study, we adhered to a method that applies limitations within physiological ranges for kinetic parameter estimation, without resorting to manual outlier adjustments to handle noisy data.

The additional Gaussian blurring with kernels of different FWHM allowed the PET image resolution effect on the IDIF to be estimated. The diameter of the internal and common carotid artery is relatively small, [5.11 ± 0.87 mm and 6.52 ± 0.98 mm in men [59]], compared to the DA (up to 27mm). The IDIF from the DA was estimated using a 10mm diameter cylinder and should not be affected by the image resolutions that we simulated. When the spatial resolution was set to a FWHM = 3.83 mm or 4 mm we observed a 5% error for the AUC of the extracted IDIF compared to the reference AIF (Fig. 5). However, the error increased markedly at lower PET resolutions. Figure 6 shows that more voxels were identified as vascular when the image resolution decreased. Presumably this reflects a greater signal spill out from vascular to neighbouring voxels and implies that partial volume correction will become necessary [60] at lower PET image resolutions to maintain an error below 5%.

Image resolution dependence also occurs with techniques that manually segment the internal carotid artery. In a recent study [2], the IDIF was investigated using manual delineation of the internal carotid arteries in the brain and compared to those of the large blood pools in the cardiac region. In the study, there was observed a strong correlation between the amplitudes of the peaks and tails of the input functions obtained from the ascending aorta, descending aorta, left ventricle, and left atrium. However, the input function derived from the carotid arteries exhibited a significant underestimation of the area under the curve ($AU{C}_{error}$ ≈ − 30%) due to unaddressed partial volume effects. We believe the ROIs are more prone to partial volume effects, as each voxel was not evaluated separately, and the resulting ROIs are affected by spill-in and spill-out from neighbouring voxels. In contrast our automated framework examines each voxel from brain images separately and tries to select voxels with minimum partial volume effects by applying the thresholding and filtering criteria. In a similar dataset, our approach achieved excellent agreement between IDIF from the brain and that from the DA ($AU{C}_{error}$ ≈− 1.59 ± 2.93%).

Other methods for arterial segmentation such as MR- and atlas-based segmentation methods can suffer from co-registration errors and inability to capture subject-specific variations [15]. Using population-based input functions [8] as an alternative method involves determining an appropriate scaling factor from images of large blood pools, arterial or venous blood sampling [61], or other factors [45] to scale the input function template for each patient. This approach adds complexity and may introduce potential sources of error. Our method combines automated clustering and thresholding of brain image data and the combination of arterial and venous time activity curves and does not require a pre-defined atlas, arterial segmentation on MRI or scaling factors.

Other methods of direct carotid segmentation on PET images select a limited number of ‘hot’ voxels within the carotid artery using an operator-selected region-of-interest [32] or techniques such as k-means clustering [32], independent component analysis [62], analysis of local minima [30], or graph-based Mumford-Shah energy-minimisation algorithms [63]. Some previously published automated segmentation methods require peripheral blood samples to adjust the estimated arterial input function [30, 62]. Some methods also require manual selection of regions of interest in the estimation process [32], while others are not easily implemented [63]. These limitations may affect the generalizability and practicality of previously described methods. While these studies have reported on the effects of errors in AIF estimation on ${K}_{i}$ estimation, they have not examined effects on the accuracy of microparameter estimates (${K}_{1}$, ${k}_{2}$, ${k}_{3}$) as we do in this study.

As an alternative approach for IDIF using a standard field-of-view scanner, whole-body dynamic PET is employed, commencing with an early cardiac scan to capture the AIF peak. Subsequently, data are collected through multiple whole-body bed passes, enabling kinetic modeling via linear Patlak analysis to estimate the net uptake rate (${K}_{i}$) [58, 64–66]. Extracting IDIF from large blood pools through the cardiac scan is advantageous, being less susceptible to partial volume effects and spill in/out [2, 14–17]. However, precise estimation of kinetic parameters ${K}_{1}$, ${k}_{2}$ and ${k}_{3}$ relies on tissue time-activity curves from early measurements, which is not possible in current whole-body ^18^F-FDG PET/CT scanning. This approach primarily determines the net influx rate (${K}_{i}$) [55, 67]. Our automated framework estimates IDIF from brain images, facilitating non-invasive AIF estimation in standard field-of-view scanners, without the need for individual partial volume effects correction. This enables mapping of ${K}_{1}$, ${k}_{2}$, ${k}_{3}$ and ${K}_{i}$.

In long axial field-of-view dynamic PET imaging, random shifts and deformations can cause non-uniform intensity changes in the human body [68]. Patient movement during prolonged scans poses challenges for visual quality and quantification accuracy, especially when estimating kinetic parameters [68, 69]. Sequential pairwise registration is recommended for dynamic PET studies [68], and a recent deep learning approach addresses motion correction in this context [70]. Although our study did not utilize motion correction, we carefully examined patient dynamic data, focusing on the last 10 min to exclude data affected by significant head movement. Additionally, we visually assessed the descending aorta volume for precise tail delineation and manual ROI positioning. In future studies, including our ongoing investigation, we aim to evaluate the impact of motion correction algorithms on kinetic parameter estimation and the accuracy of extracting IDIF from descending aorta and brain PET data.

Our study presents an automated framework for IDIF estimation from brain images using dynamic long axial field-of-view data from the Biograph Quadra Vision PET scanner. The framework was compared with the IDIF from a large blood pool (DA) method using total body PET ^18^F-FDG. Future studies should aim to validate our proposed framework against the gold standard arterial blood sampling to further evaluate its accuracy and reliability.

The automated framework proposed here estimates IDIF from brain PET images, requiring a spatial resolution greater than FWHM = 4mm for precise thresholding. Future research aims to validate this approach with images from various PET systems. The count-rate sensitivity gap between the long axial field-of-view scanner in this study and standard axial field-of-view PET systems indicates a potential for increased voxel-wise noise. This highlights a critical area for future studies, addressing both spatial resolution and voxel-wise noise in the automated framework for IDIF estimation from brain images.

In the irreversible 2TCM defined in Eq. (3), we did not distinguish between plasma and whole blood concentrations of ^18^F-FDG. Previous reports [71, 72] have noted a systematic difference between these concentrations in humans. While unlikely, it may bias parameter estimates by around 5–10% [45].

The proposed automated AIF extraction, tested on 12 oncological patients (with 6 used for validation), demonstrated initial feasibility. Acknowledging the limitation of a small sample size and range of pathologies, future studies should include a broader range of disorders and therapeutic interventions in which plasma clearance and the shape of the AIF may be altered, including patients with diabetes or undergoing oncologic therapy.

An IDIF approach is limited by patient motion. In this study, we visually assessed the descending aorta volume, especially in later frames for accuracy. However, patient head movement can shift ROIs affecting the carotid and venous input function. To counter this, using a frame-by-frame motion correction algorithm [73, 74] is advised. Metabolite correction was omitted in this study due to its insignificance in ^18^F-FDG studies [15]. Arterial blood sampling yields an AIF that differs from the AIF in the descending aorta due to dispersion effects. The peak activity in the aorta appears earlier, higher, and narrower than the peak from arterial cannulation. However, it shows minor variation in the AUC [46]. It is anticipated that dispersion-induced changes are negligible between the descending aorta and the arterial vasculature AIF in the brain. Nonetheless, we observed slight disparities in peak heights attributed to noise and the duration of sampling.

## Conclusion

The study introduces an automated framework for precise estimation of the image-derived input function from ^18^F-FDG-PET brain images, which eliminates the requirement for additional partial volume effect correction. The framework decreases operator-dependency and enhances the potential of parametric PET adoption in clinical settings using high-resolution PET systems. The study suggests combining voxels identified as being from brain arteries or veins can be combined to minimize errors in an image-derived input function.

## Supplementary Information

**Additional file 1**. **Table S1**. Patient demographics summary. **Table S2**. Shows comparison of averaged clustered IDIFs with $\textit{ID}_{\textit{DA}} IF$ and $\textit{AUC}_{\textit{errors}}$ for each cluster at various threshold levels (a1 = 0.1 to 0.9 when a2 = 0.9). **Table S3**. Comparison of $\textit{AUC}_{\textit{errors}}$ for time periods (TP1 to TP7) between IDIFs derived from the descending aorta, brain arteries, and brain veins for each patient (P1 to P6). **Supplementary Table 4**. Kinetic parameters estimates from gray and white matter (P1 to P6).

**Additional file 2**. **Fig. S1** Voxel-wise Bland–Altman plots illustrate the percentage differences in $\textit{K}_{1}$, $\textit{k}_{2}$, and $\textit{k}_{3}$ for subjects P7-P12 in the validation cohort. These plots compare parametric maps obtained using the descending aorta IDIF (${\text{IDIF}}_{{{\text{DA}}}}$) and the automatically extracted image-derived input function from brain images (${\text{IDIF}}_{{{\text{Auto}}}}$).

**Additional file 3**. **Fig. S2** Voxel-wise Bland–Altman plots for $2TCM K_{i}$ and $Patlak K_{i}$ (subjects P7–P12 in the validation cohort) depict percentage differences between parametric maps using the descending aorta IDIF ($IDIF_{DA}$) and the automatically extracted image-derived input function from brain images ($IDIF_{Auto}$).

**Additional file 4Fig. S3** Scatter plots showing the mean values for both GM and WM across $K_{1}$, $k_{2}$, $k_{3}$ and $K_{i}$ for both 2TCM and Patlak for the six subjects in the validation cohort (subjects P7–P12). The plots indicate the coefficient of determination ($R^{2}$) and slope with 95% confidence intervals for the correlation between the parametric maps obtained using the descending aorta IDIF ($IDIF_{DA}$) and the automatically extracted image-derived input function from brain images ($IDIF_{Auto}$) showing variations in the $R^{2}$ and slopes of individual rate constant estimation.

## Data Availability

The data supporting our findings are available from reference [2], but they are not publicly accessible due to licensing restrictions.

## Abbreviations

AIF: Arterial input function
IDIF: Image-derived input function
PET: Positron emission tomography
^18^F-FDG: ^18^F-fluorodeoxyglucose
2TCM: 2 Tissue compartment model
ROI: Region of interest
LSTM: Long short-term memory
FWHM: Full-width at half-maximum
PSF: Point spread function
ToF: Time-of-flight
DA: Descending aorta
$\textit{IDIF}_{\textit{DA}}$: Image-derived input function from DA
$\textit{IDIF}_{\textit{Auto}}$: Automated brain IDIF
$\textit{IDIF}_{\textit{Artery}}$: Image-derived arterial
$\textit{IDIF}_{\textit{Vein}}$: Venous input functions
$\textit{AUC}$: Area under the curve
$\textit{NRMSE}$: Root mean square error
TTAC: Tissue time activity curve
