# Supplementary material for: Automated extraction of the arterial input function from brain images for parametric PET studies
Source: EJNMMI Res. 2024 Apr 1;14:33. doi: 10.1186/s13550-024-01100-x (PMC11372015; doi:10.1186/s13550-024-01100-x)
Supplement: Supplementary file 1 — Additional file 1. Table S1. Patient demographics summary. Table S2. Shows comparison of averaged clustered IDIFs with $\textit{ID}_{\textit{DA}} IF$IDDAIF and $\textit{AUC}_{\textit{errors}}$AUCerrors for each cluster at various threshold levels (a1 = 0.1 to 0.9 when a2 = 0.9). Table S3. Comparison of $\textit{AUC}_{\textit{errors}}$AUCerrors for time periods (TP1 to TP7) between IDIFs derived from the descending aorta, brain arteries, and brain veins for each patient (P1 to P6). Supplementary Table 4. Kinetic parameters estimates from gray and white matter (P1 to P6). [file 13550_2024_1100_MOESM1_ESM.docx]

**Automated Extraction of the Arterial Input Function from Brain Images for Parametric PET Studies**

Hamed Moradi, ^1, 2, 3^ Rajat Vashistha,^1, 2^ Soumen Ghosh,^1, 2^ Kieran O'Brien,^1, 2, 3^ Amanda Hammond,^2, 3^ Axel Rominger,^4^ Hasan Sari,^4,5^ Kuangyu Shi,^4^ Viktor Vegh,^1, 2^ and David Reutens^1,2^

^1^Centre for Advanced Imaging, [Australian Institute for Bioengineering and Nanotechnology](https://aibn.uq.edu.au/), The University of Queensland, Brisbane, Australia

^2^ARC Training Centre for Innovation in Biomedical Imaging Technology, The University of Queensland, Brisbane, Australia

^3^Siemens Healthcare Pty Ltd, Melbourne, Australia

^4^Department of Nuclear Medicine, Inselspital, Bern University Hospital, University of Bern, Freiburgstrasse 18, 3010 Bern, Switzerland

^5^Advanced Clinical Imaging Technology, Siemens Healthcare AG, Lausanne, Switzerland

**Corresponding Author:** Viktor Vegh

Centre for Advanced Imaging

[Australian Institute for Bioengineering and Nanotechnology](https://aibn.uq.edu.au/)

The University of Queensland

Brisbane, Australia

Email: [v.vegh@uq.edu.au](mailto:v.vegh@uq.edu.au), [viktor.vegh@cai.uq.edu.au](mailto:viktor.vegh@cai.uq.edu.au)

Supplementary Table 1 Patient demographics summary.

| Group | Subject | Age (years) | Sex | Weight (kg) | Injected dose (MBq) | Blood glucose (µmol·mL−1) | Diagnosis |
| --- | --- | --- | --- | --- | --- | --- | --- |
| Model adjustment | P1 | 80 | Female | 94 | 274.5 | 6.3 | Fallopian tube |
|  | P2 | 77 | Male | 81 | 261.0 | 5.9 | Lymphoma |
|  | P3 | 29 | Male | 130 | 398.0 | 6.6 | Lymphoma |
|  | P4 | 66 | Female | 52 | 164.0 | 5.1 | Breast |
|  | P5 | 48 | Male | 85 | 276.0 | 5.9 | Lymphoma |
|  | P6 | 68 | Male | 79 | 227.0 | 9.5 | Lymphoma |
| Validation | P7 | 75 | Female | 65 | 190.0 | 5.7 | Breast |
|  | P8 | 46 | Female | 70 | 217.0 | 6.8 | Breast |
|  | P9 | 70 | Female | 53 | 168.0 | 6.8 | Lung |
|  | P10 | 39 | Female | 57 | 178.0 | 5.7 | Breast |
|  | P11 | 62 | Male | 70 | 213.0 | 5.8 | Lymphoma |
|  | P12 | 85 | Male | 78 | 248.7 | 5.3 | Lymphoma |

Supplementary Table 2 shows comparison of averaged clustered IDIFs with ${IDIF}_{DA}$ and ${AUC}_{errors}$ for each cluster at various threshold levels ($a1=0.1 to 0.9$ when $a2=0.9$).

|  | a1 | 0.1 | 0.2 | 0.3 | 0.4 | 0.5 | 0.6 | 0.7 | 0.8 | 0.9 | Average (0.4 to 0.6) |
| --- | --- | --- | --- | --- | --- | --- | --- | --- | --- | --- | --- |
| P1 | Artery | -13.48 | -21.40 | -21.08 | -22.89 | -23.71 | -24.61 | -27.47 | -28.87 | -30.56 | -22.27 |
|  | Vein | -7.80 | -8.69 | -9.93 | -3.86 | -4.45 | -6.11 | -7.63 | -9.11 | -13.31 | -4.94 |
| P2 | Artery | -100.00 | -18.60 | -16.69 | -10.68 | -18.81 | -17.78 | -19.75 | -20.51 | -20.12 | -16.40 |
|  | Vein | -13.73 | -14.57 | -12.99 | -8.44 | -8.21 | -7.03 | -10.43 | -11.73 | -13.23 | -7.35 |
| P3 | Artery | -17.47 | -20.20 | -22.74 | -22.92 | -20.45 | -20.93 | -20.98 | -20.59 | -17.71 | -20.38 |
|  | Vein | -8.28 | -1.84 | -3.31 | 3.46 | 6.52 | 7.00 | -6.53 | -5.96 | 5.31 | 5.54 |
| P4 | Artery | -1.22 | -8.75 | -9.41 | -7.42 | -13.64 | -16.03 | -19.86 | -20.75 | -20.73 | -12.99 |
|  | Vein | -3.27 | -11.75 | -9.36 | -12.37 | -9.30 | -12.47 | -15.78 | -17.07 | -18.86 | -11.58 |
| P5 | Artery | -100.00 | -25.11 | -24.82 | -22.14 | -24.51 | -25.94 | -28.82 | -29.54 | -28.83 | -24.74 |
|  | Vein | -4.46 | -6.25 | -6.89 | 4.63 | -3.42 | -6.13 | -9.08 | -10.25 | -9.63 | -2.19 |
| P6 | Artery | -100.00 | -19.82 | -18.15 | -12.19 | -16.56 | -17.32 | -20.20 | -22.29 | -26.54 | -15.77 |
|  | Vein | -11.95 | -7.01 | -5.58 | 4.71 | 3.53 | 1.92 | -1.94 | -3.57 | -8.01 | 2.70 |
| Mean | Artery | -55.36 | -18.98 | -18.82 | -16.37 | -19.61 | -20.44 | -22.85 | -23.76 | -24.08 | -18.76 |
|  | Vein | -8.25 | -8.35 | -8.01 | -1.98 | -2.55 | -3.80 | -8.57 | -9.62 | -9.62 | -2.97 |
| SD | Artery | 49.19 | 5.48 | 5.48 | 7.05 | 4.17 | 4.10 | 4.15 | 4.27 | 5.26 | 4.44 |
|  | Vein | 4.08 | 4.45 | 3.45 | 7.37 | 6.34 | 7.01 | 4.58 | 4.70 | 8.22 | 6.36 |

Supplementary Table 3 Comparison of ${AUC}_{errors}$ for time periods (TP1 to TP7) between IDIFs derived from the descending aorta, brain arteries, and brain veins for each patient (P1 to P6)

|  |  | T1 | T2 | T3 | T4 | T5 | T6 | T7 |
| --- | --- | --- | --- | --- | --- | --- | --- | --- |
| P1 | ${{IDIF}_{Artery}}_{0.4-0.6}$ | 9.00 | -25.39 | -30.43 | -27.85 | -25.59 | -21.28 | -19.66 |
|  | ${{IDIF}_{Vein}}_{0.4-0.6}$ | 25.32 | -0.34 | -12.75 | -9.42 | -11.48 | -3.69 | 1.11 |
| P2 | ${{IDIF}_{Artery}}_{0.4-0.6}$ | 8.03 | -14.81 | -16.78 | -19.92 | -22.48 | -26.52 | -23.88 |
|  | ${{IDIF}_{Vein}}_{0.4-0.6}$ | 20.61 | -3.69 | -19.07 | -21.51 | -16.02 | -10.09 | -5.09 |
| P3 | ${{IDIF}_{Artery}}_{0.4-0.6}$ | -11.97 | -28.90 | -26.40 | -20.84 | -21.26 | -15.73 | -9.92 |
|  | ${{IDIF}_{Vein}}_{0.4-0.6}$ | 20.80 | 0.80 | -0.05 | -5.41 | -7.72 | 17.68 | 32.64 |
| P4 | ${{IDIF}_{Artery}}_{0.4-0.6}$ | -10.93 | -17.24 | -14.13 | -12.26 | -12.00 | -9.77 | -10.11 |
|  | ${{IDIF}_{Vein}}_{0.4-0.6}$ | -3.23 | -8.43 | -12.72 | -11.61 | -16.24 | -14.36 | -10.67 |
| P5 | ${{IDIF}_{Artery}}_{0.4-0.6}$ | -9.62 | -29.32 | -27.01 | -24.10 | -24.82 | -23.23 | -18.97 |
|  | ${{IDIF}_{Vein}}_{0.4-0.6}$ | -7.60 | -18.41 | -19.96 | -4.43 | -1.10 | 25.54 | 32.32 |
| P6 | ${{IDIF}_{Artery}}_{0.4-0.6}$ | 7.31 | -19.28 | -13.38 | -17.57 | -23.43 | -18.17 | -8.04 |
|  | ${{IDIF}_{Vein}}_{0.4-0.6}$ | 39.15 | 7.25 | 3.08 | -14.16 | -1.63 | 2.36 | 6.87 |
| Mean | ${{IDIF}_{Artery}}_{0.4-0.6}$ | -1.36 | -22.49 | -21.36 | -20.42 | -21.60 | -19.12 | -15.10 |
|  | ${{IDIF}_{Vein}}_{0.4-0.6}$ | 15.84 | -3.80 | -10.25 | -11.09 | -9.03 | 2.91 | 9.53 |
| SD | ${{IDIF}_{Artery}}_{0.4-0.6}$ | 9.51 | 5.67 | 6.79 | 4.90 | 4.52 | 5.42 | 5.98 |
|  | ${{IDIF}_{Vein}}_{0.4-0.6}$ | 16.30 | 8.06 | 8.81 | 5.74 | 6.14 | 14.38 | 17.09 |

|  |  | 2TCM | | | | | | | | | | Patlak | |
| --- | --- | --- | --- | --- | --- | --- | --- | --- | --- | --- | --- | --- | --- |
|  |  | K1 | | k2 | | k3 | | Vb | | Ki | | Ki | |
|  |  | GM | WM | GM | WM | GM | WM | GM | WM | GM | WM | GM | WM |
| P7 | ${IDIF}_{DA}$ | 0.193±0.054 | 0.068±0.017 | 0.134±0.039 | 0.096±0.039 | 0.061±0.015 | 0.027±0.017 | 0.019±0.017 | 0.016±0.009 | 0.058±0.007 | 0.014±0.004 | 0.054±0.007 | 0.007±0.015 |
|  | ${IDIF}_{Auto}$ | 0.193±0.053 | 0.063±0.016 | 0.133±0.038 | 0.084±0.036 | 0.062±0.015 | 0.022±0.017 | 0.017±0.016 | 0.018±0.010 | 0.059±0.008 | 0.013±0.005 | 0.053±0.008 | 0.008±0.014 |
|  | Error (%) | -0.23 | -7.83 | -1.24 | -20.13 | 1.87 | -18.57 | -10.40 | 17.70 | 2.05 | -9.45 | -1.78 | -6.87 |
| P8 | ${IDIF}_{DA}$ | 0.204±0.045 | 0.065±0.015 | 0.165±0.062 | 0.086±0.034 | 0.047±0.014 | 0.018±0.011 | 0.033±0.019 | 0.023±0.013 | 0.045±0.007 | 0.010±0.004 | 0.042±0.007 | 0.007±0.012 |
|  | ${IDIF}_{Auto}$ | 0.174±0.039 | 0.055±0.013 | 0.158±0.076 | 0.077±0.037 | 0.058±0.019 | 0.020±0.012 | 0.041±0.021 | 0.026±0.013 | 0.047±0.007 | 0.011±0.004 | 0.044±0.007 | 0.007±0.012 |
|  | Error (%) | -15.02 | -14.72 | -4.41 | -10.84 | 21.84 | 19.13 | 26.17 | 11.56 | 3.42 | 8.05 | 2.65 | 5.15 |
| P9 | ${IDIF}_{DA}$ | 0.178±0.046 | 0.083±0.041 | 0.174±0.065 | 0.143±0.102 | 0.035±0.010 | 0.020±0.018 | 0.040±0.019 | 0.028±0.026 | 0.029±0.005 | 0.009±0.003 | 0.027±0.004 | 0.004±0.009 |
|  | ${IDIF}_{Auto}$ | 0.150±0.041 | 0.069±0.034 | 0.154±0.070 | 0.123±0.096 | 0.040±0.013 | 0.023±0.019 | 0.054±0.022 | 0.032±0.026 | 0.031±0.005 | 0.010±0.003 | 0.028±0.004 | 0.004±0.009 |
|  | Error (%) | -16.05 | -17.27 | -11.33 | -14.43 | 15.50 | 14.68 | 35.16 | 14.95 | 4.85 | 5.78 | 1.99 | 2.49 |
| P10 | ${IDIF}_{DA}$ | 0.171±0.030 | 0.067±0.021 | 0.117±0.022 | 0.092±0.036 | 0.043±0.009 | 0.027±0.010 | 0.039±0.027 | 0.031±0.017 | 0.045±0.006 | 0.015±0.004 | 0.042±0.005 | 0.005±0.015 |
|  | ${IDIF}_{Auto}$ | 0.182±0.033 | 0.069±0.023 | 0.125±0.028 | 0.088±0.044 | 0.050±0.011 | 0.026±0.011 | 0.041±0.025 | 0.033±0.017 | 0.047±0.006 | 0.016±0.004 | 0.046±0.006 | 0.006±0.016 |
|  | Error (%) | 6.29 | 2.73 | 6.95 | -3.52 | 15.95 | 6.36 | 5.72 | 5.41 | 4.44 | 6.60 | 9.03 | 8.14 |
| P11 | ${IDIF}_{DA}$ | 0.143±0.034 | 0.040±0.019 | 0.125±0.056 | 0.101±0.058 | 0.054±0.019 | 0.037±0.030 | 0.044±0.025 | 0.010±0.009 | 0.043±0.008 | 0.008±0.004 | 0.041±0.008 | 0.008±0.009 |
|  | ${IDIF}_{Auto}$ | 0.163±0.038 | 0.042±0.020 | 0.121±0.046 | 0.085±0.051 | 0.046±0.019 | 0.025±0.028 | 0.036±0.025 | 0.012±0.010 | 0.043±0.009 | 0.007±0.004 | 0.039±0.009 | 0.009±0.008 |
|  | Error (%) | 13.54 | 4.19 | -2.92 | -16.15 | -15.82 | -26.82 | -17.98 | 13.38 | 0.42 | -6.26 | -3.89 | 6.71 |
| P12 | ${IDIF}_{DA}$ | 0.156±0.041 | 0.045±0.020 | 0.112±0.028 | 0.064±0.037 | 0.069±0.017 | 0.025±0.024 | 0.006±0.008 | 0.005±0.006 | 0.057±0.009 | 0.009±0.004 | 0.056±0.009 | 0.009±0.010 |
|  | ${IDIF}_{Auto}$ | 0.167±0.030 | 0.052±0.012 | 0.103±0.060 | 0.058±0.015 | 0.062±0.016 | 0.021±0.004 | 0.022±0.020 | 0.008±0.011 | 0.058±0.003 | 0.008±0.003 | 0.058±0.003 | 0.010±0.007 |
|  | Error (%) | 7.05 | 15.56 | -8.04 | -9.38 | -10.14 | -16.03 | -41.10 | -55.16 | 1.76 | -6.57 | 3.58 | 6.39 |
| Average | ${IDIF}_{DA}$ | 0.173±0.039 | 0.061±0.021 | 0.137±0.054 | 0.094±0.049 | 0.053±0.015 | 0.025±0.015 | 0.026±0.021 | 0.022±0.018 | 0.048±0.006 | 0.011±0.004 | 0.045±0.006 | 0.007±0.011 |
|  | ${IDIF}_{Auto}$ | 0.173±0.041 | 0.059±0.021 | 0.134±0.044 | 0.089±0.048 | 0.051±0.015 | 0.023±0.018 | 0.028±0.025 | 0.024±0.019 | 0.047±0.007 | 0.011±0.004 | 0.043±0.007 | 0.007±0.011 |
|  | Error (%) | -0.7±12.3 | -2.9±12.6 | -3.5±6.3 | -12.4±5.8 | 4.9±15.4 | -3.5±19.3 | -0.4±28.5 | 1.3±28.0 | 2.8±1.7 | -0.3±7.9 | 1.9±4.5 | 3.7±5.5 |

Supplementary Table 4 Kinetic parameters estimates from gray and white matter (P7 to P12)
