# Supplementary figures and images for: Automated extraction of the arterial input function from brain images for parametric PET studies
Source: EJNMMI Res. 2024 Apr 1;14:33. doi: 10.1186/s13550-024-01100-x (PMC11372015; doi:10.1186/s13550-024-01100-x)

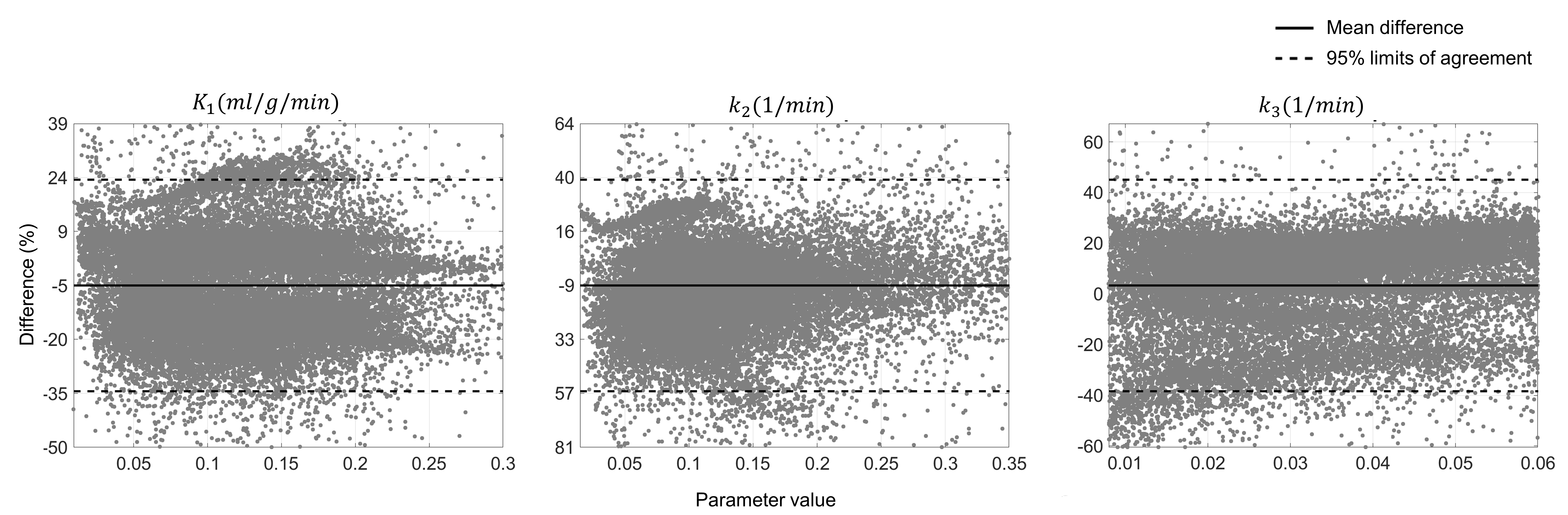

Supplement: Supplementary file 2 — Additional file 2. Fig. S1 Voxel-wise Bland–Altman plots illustrate the percentage differences in $\textit{K}_{1}$K1, $\textit{k}_{2}$k2, and $\textit{k}_{3}$k3 for subjects P7-P12 in the validation cohort. These plots compare parametric maps obtained using the descending aorta IDIF (${\text{IDIF}}_{{{\text{DA}}}}$IDIFDA) and the automatically extracted image-derived input function from brain images (${\text{IDIF}}_{{{\text{Auto}}}}$IDIFAuto). [file 13550_2024_1100_MOESM2_ESM.png]

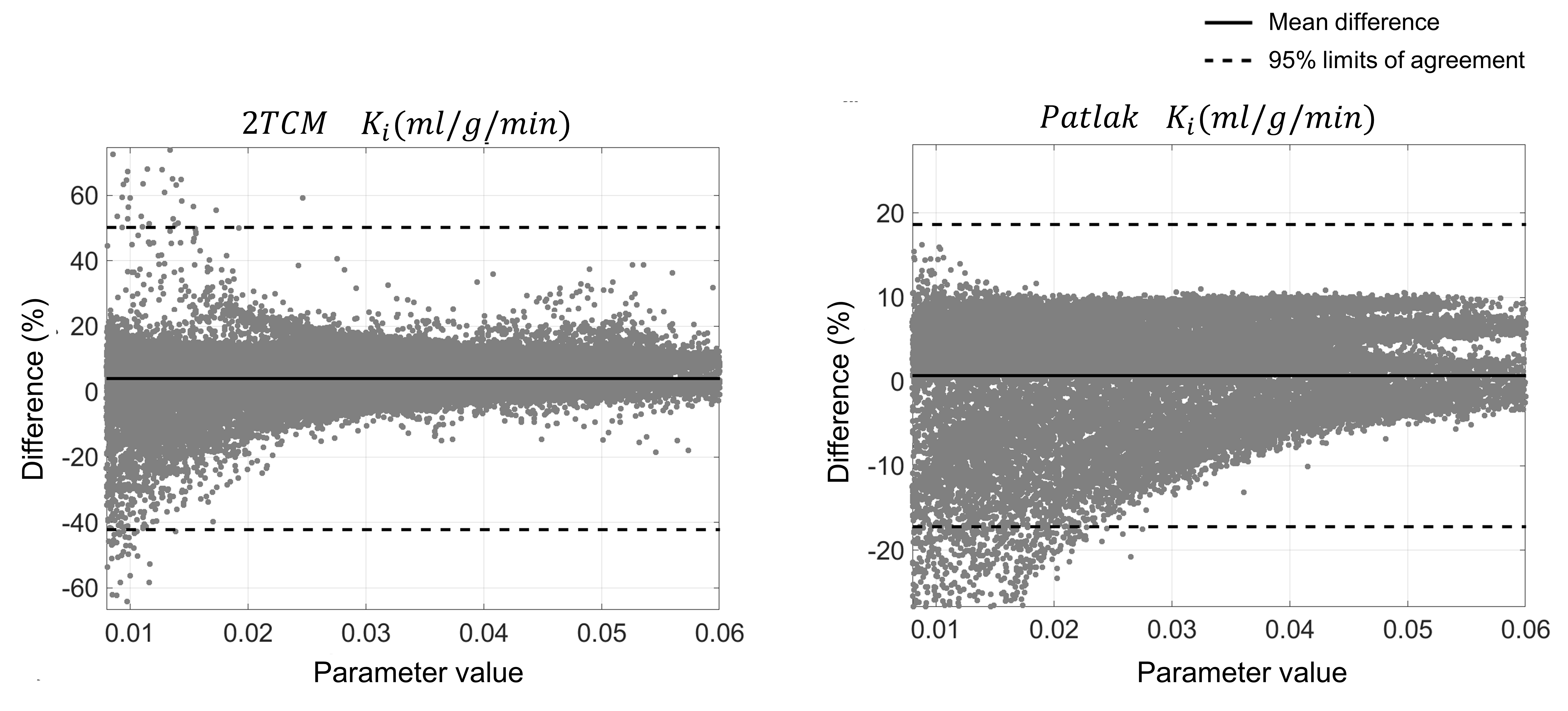

Supplement: Supplementary file 3 — Additional file 3. Fig. S2 Voxel-wise Bland–Altman plots for $2TCM K_{i}$2TCMKi and $Patlak K_{i}$PatlakKi (subjects P7–P12 in the validation cohort) depict percentage differences between parametric maps using the descending aorta IDIF ($IDIF_{DA}$IDIFDA) and the automatically extracted image-derived input function from brain images ($IDIF_{Auto}$IDIFAuto). [file 13550_2024_1100_MOESM3_ESM.png]

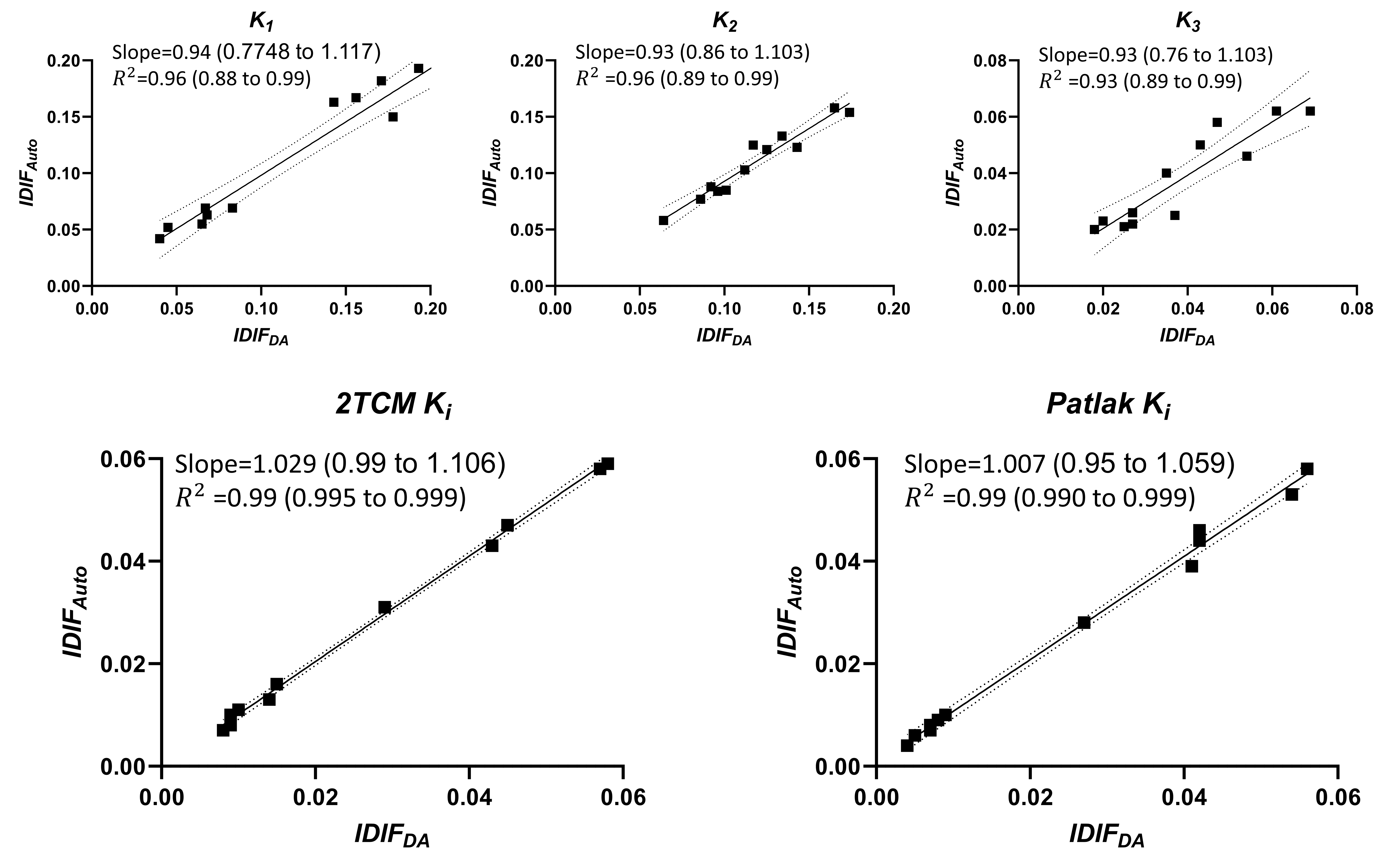

Supplement: Supplementary file 4 — Additional file 4Fig. S3 Scatter plots showing the mean values for both GM and WM across $K_{1}$K1, $k_{2}$k2, $k_{3}$k3 and $K_{i}$Ki for both 2TCM and Patlak for the six subjects in the validation cohort (subjects P7–P12). The plots indicate the coefficient of determination ($R^{2}$R2) and slope with 95% confidence intervals for the correlation between the parametric maps obtained using the descending aorta IDIF ($IDIF_{DA}$IDIFDA) and the automatically extracted image-derived input function from brain images ( [file 13550_2024_1100_MOESM4_ESM.png]
